# Ultrasound‐Activated Precise Sono‐Immunotherapy for Breast Cancer with Reduced Pulmonary Fibrosis

**DOI:** 10.1002/advs.202407609

**Published:** 2024-12-16

**Authors:** Xiang Li, Gao He, Hui Jin, Xinyu Xiang, Dong Li, Renmiao Peng, Jing Tao, Xinping Li, Kaiyang Wang, Yu Luo, Xiaoan Liu

**Affiliations:** ^1^ Department of Thyroid‐Breast Surgery The Fourth Affiliated Hospital of Nanjing Medical University 298 Nanpu Road Nanjing Jiangsu 210032 P. R. China; ^2^ Breast Disease Center The First Affiliated Hospital of Nanjing Medical University 300 Guangzhou Road Nanjing Jiangsu 210029 P. R. China; ^3^ The Afffliated Taizhou People's Hospital of Nanjing Medical University Taizhou School of Clinical Medicine Nanjing Medical University 366 Taihu Road Taizhou Jiangsu 225300 P. R. China; ^4^ Department of Breast surgery The Affiliated Tumor Hospital of Nantong University 30 Tongyang north road Nantong Jiangsu 226361 P. R. China; ^5^ Shanghai Engineering Research Center of Pharmaceutical Intelligent Equipment Shanghai Frontiers Science Research Center for Druggability of Cardiovascular Non‐coding RNA Institute for Frontier Medical Technology School of Chemistry and Chemical Engineering Shanghai University of Engineering Science Shanghai 201620 P. R. China

**Keywords:** breast cancer, dendritic mesoporous silica, pulmonary fibrosis, sono‐immunotherapy

## Abstract

Immune checkpoint inhibitors have demonstrated remarkable efficacy across various cancer types. However, immune‐related adverse events (irAEs) pose a significant challenge in immunotherapy, particularly the associated pneumonia as the primary adverse reaction, which can lead to irreversible pulmonary fibrosis. Additionally, monotherapy with programmed death ligand (PD‐L1) inhibitors has shown limited effectiveness. Therefore, to improve the response rate of immunotherapy and reduce pulmonary fibrosis, this study designed and prepared an intelligent nanodrug based on dendritic mesoporous silica nanoparticles (DMSNs) loaded with a sono‐sensitive agent protoporphyrin IX (PpIX). Additionally, a reactive oxygen species (ROS) sensitive linker is used to attach the immunotherapeutic drug PD‐L1 inhibitor (aPD‐L1) to DMSNs via covalent bonds. The external ultrasound (US) activates PpIX to generate ROS, which breaks the linker to release aPD‐L1 to induce sonodynamic therapy (SDT) and immunotherapy. This sono‐immnotherapy approach demonstrated excellent outcomes in tumor inhibition, eliciting immune responses, and reducing pulmonary fibrosis. Overall, this study offers a new, efficient, and safe method for breast cancer treatment, and expands the application of immunotherapy.

## Graphical abstract

This study introduces an ultrasound‐activated nanodrug, DMSNs‐PpIX‐aPD‐L1, for precise breast cancer treatment. It uses ultrasound to generate reactive oxygen species, inducing immunogenic cell death, and releasing aPD‐L1. This approach addresses triple‐negative breast cancer challenges, reducing recurrence, metastasis, and pulmonary fibrosis, enhancing treatment efficacy and safety.

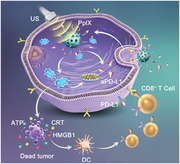

## 1 Introduction

Breast cancer has the highest incidence and is the second leading cause of death among female malignant tumors, seriously affecting women's physical and mental health.^[^ ^1^, ^2^, ^3^ ^]^ In particular, the complexity of triple‐negative breast cancer (TNBC) necessitates a multifaceted approach to treatment. Despite advancements in cancer treatment strategies, including surgery, chemotherapy, and radiation therapy, the recurrence and metastasis of TNBC remain significant challenges, underscoring the urgent need for innovative treatment modalities.^[^ ^4^, ^5^ ^]^

Recent advances in immunotherapy, which harness the human immune system to target cancer cells, have emerged as effective cancer treatments.^[^ ^6^, ^7^, ^8^ ^]^ However, due to unregulated tissue accumulation and the inherent biological activity of these drugs, immune related adverse events (irAEs) pose significant challenges in immunotherapy, especially immune checkpoint inhibitor‐associated pneumonia as the primary adverse reaction in TNBC. Pneumonia can lead to irreversible pulmonary fibrosis, and, given the prolonged survival of breast cancer patients, these adverse reactions may be further exacerbated.^[^ ^9^, ^10^, ^11^ ^]^

Biological thiols on the surface of activated T cells may provide a localized target for immunotherapy.^[^ ^9^, ^12^ ^]^ Additionally, nanoclusters have been designed to release programmed cell death protein 1 (PD‐1) antibodies in the TME,^[^ ^13^, ^14^, ^15^ ^]^ enhancing the efficacy of adoptive T cell cancer therapy while reducing its side effects.^[^ ^16^, ^17^ ^]^ However, these immune stimulators rely on endogenous stimuli, which may not be sufficient to enable a controllable release of the drug. Therefore, the creation of immunotherapies with high spatial and temporal activation precision could enhance treatment effectiveness and reduce immune‐related side effects, significantly supporting the advancement and utilization of immunotherapy.^[^ ^18^, ^19^, ^20^ ^]^

Ultrasound (US) can penetrate tissues over 10 cm, making it effective for treating deep tumors like breast cancer.^[^ ^21^, ^22^, ^23^, ^24^, ^25^ ^]^ Notably, sonodynamic therapy (SDT) is recognized for its non‐invasive and deep tissue penetration compared to light‐based therapies.^[^ ^26^, ^27^, ^28^, ^29^, ^30^, ^31^ ^]^ In SDT, sono‐sensitizers generate reactive oxygen species (ROS). These ROS break ROS‐sensitive linkers to release drugs as needed, enhancing the stabilization of therapeutic agents.^[^ ^32^, ^33^, ^34^, ^35^ ^]^ Additionally, SDT can induce immunogenic cell death (ICD). Cells undergoing ICD emit stress signals through endogenous molecules called damage‐associated molecular patterns (DAMPs), including adenosine triphosphate (ATP), high mobility group box 1 (HMGB1), and calreticulin (CRT). These signals are detected by antigen‐presenting cells, leading to adaptive immune responses driven by cytotoxic T lymphocytes (CTLs) that further enhance the efficacy of immune checkpoint inhibitors.^[^ ^36^, ^37^, ^38^ ^]^

**Scheme 1:**
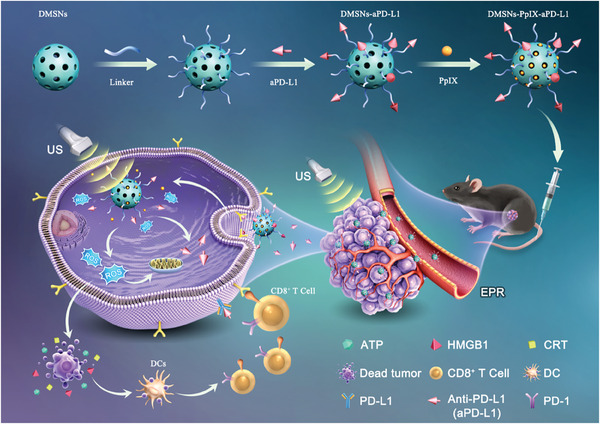
Schematic depiction of the synthesis process and the underlying therapeutic mechanism of DMSNs‐PpIX‐aPD‐L1 for cancer cells.

This study aims to address the key challenges of TNBC recurrence and metastasis by exploring innovative SDT and immunotherapy with significant practical value. Additionally, this study focuses on designing and preparing a composite structure based on a mesoporous silica drug‐controlled release system and developing precision treatment strategies for TNBC (**Scheme** **1**). First, dendritic mesoporous silica nanoparticles (DMSNs) were synthesized. Subsequently, the immunotherapeutic drug PD‐L1 inhibitor (aPD‐L1) was covalently attached to DMSNs using a ^1^O_2_‐sensitive thioacetone linker, 3,3′‐(propane‐2,2‐diylbis(sulfanediyl)) dipropionic acid (TK), forming DMSNs‐aPD‐L1. Lastly, using the ordered structure and high storage capacity of DMSNs, the hydrophobic sono‐sensitive agent protoporphyrin IX (PpIX) was loaded into the pores to obtain DMSNs‐PpIX‐aPD‐L1. Unlike traditional DMSN delivery systems, this design combines SDT and immunotherapy by loading PpIX and aPD‐L1 onto DMSNs for synergistic treatment. Upon activation by external US, DMSNs‐PpIX‐aPD‐L1 generates ROS in situ to induce ICD. Additionally, ROS cleaves the TK linker for the on‐demand release of aPD‐L1. This combination achieved controlled release of the immunotherapeutic drug while promoting ROS production. The sono‐immnotherapy improves the spatiotemporal precision of immunotherapeutic drug release and offers significant research value and practical applications in biomedicine.

## 2 Results and Discussion

**Figure 1:**
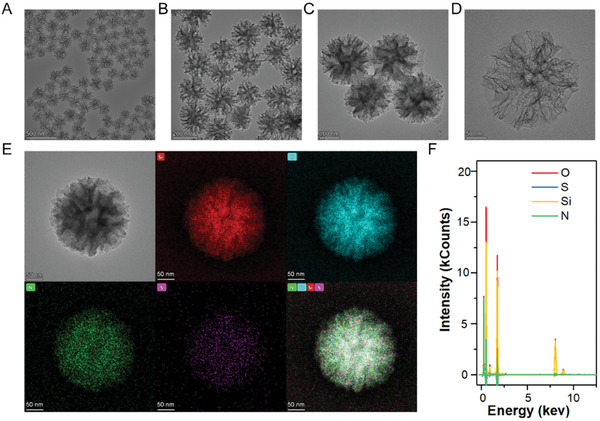
TEM images of DMSNs with various scale bars: A) 500 nm; B) 200 nm; C) 100 nm; D) 50 nm. E) Elemental mapping of DMSNs‐PpIX‐aPD‐L1. Scale bar: 50 nm F) Corresponding element intensity analysis from the elemental mapping.

DMSNs were prepared via one‐pot synthesis. The transmission electron microscopic (TEM) analysis revealed a branched spherical structure with a uniform particle size of ≈200 nm (**Figure** **1**A–D). Such a size is ideal for DMSNs to be delivered in tumors as it effectively utilizes the enhanced permeability and retention (EPR) effect. The EPR effect allows nanoparticles of certain sizes (20–250 nm) to penetrate more easily and remain in the tumor tissue for extended periods, due to tumors’ abnormal blood vessel structure and lack of efficient lymphatic drainage. Within this range, DMSNs can efficiently accumulate in tumors with longer retention, making this strategy more potent in targeting cancer cells, thereby increasing therapeutic efficacy while reducing side effects.

**Figure 2:**
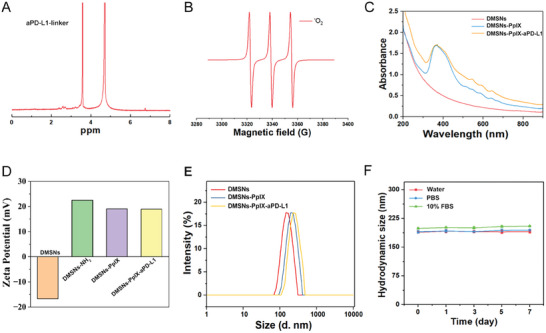
Characterization of DMSNs‐PpIX‐aPD‐L1. A) ^1^H NMR spectrum of aPD‐L1‐linker. B) ESR signal of ^1^O_2_ generated from DMSNs‐PpIX‐aPD‐L1 under US. C) UV–vis analysis. D) Zeta potential. E) Particle size distribution. F) DMSNs‐PpIX‐aPD‐L1 particle stability analysis over 7 days.

Then, DMSNs‐aPD‐L1 was synthesized by attaching the immunotherapeutic drug aPD‐L1 to DMSNs via the TK linker. The synthesis pathway is shown in Figure S1 (Supporting Information). The elemental composition of DMSNs‐aPD‐L1 was then analyzed using element mapping. Results indicated that besides the main Si and O elements in DMSNs, the nanoparticles contained a uniform distribution of N and S from aPD‐L1 and the TK linker, respectively (Figure 1E,F). As shown in Figure S2, (Supporting Information), the Fourier Transform Infrared (FTIR) spectra of the DMSNs‐PpIX‐aPD‐L1 exhibit a distinctive peak at 1654 cm⁻¹, corresponding to the C═O stretching vibration within the polypeptide backbone—a hallmark of the amide I band. This band serves as a crucial indicator of protein secondary structure. The presence of the peak suggests successful conjugation of aPD‐L1 onto DMSNs. This was further confirmed by ^1^H Nuclear magnetic resonance (NMR) (**Figure 2**A; Figure S3, Supporting Information). The connection of 3‐aminopropyltriethoxysilane (APTES) to DMSNs was confirmed by the appearance of a quartet peak between δH 3.55‐3.0, attributed to the ethoxy groups connected to oxygen in the APTES structure, during the hydrolytic coupling. In addition, the terminal methyl signals appeared as a triplet at δH 1.08‐1.05. However, due to the use of deuterium oxide (D_2_O) as the deuterated solvent, the active amino hydrogen could not be identified in the ^1^H‐NMR spectrum. Subsequently, the modification with the TK linker was confirmed by the appearance of carboxyl hydrogen protons and N‐H chemical signals between δH 10.29–10.21 in the ^1^H‐NMR spectrum, along with methylene chemical shift signals at δH 4.33 and 3.15 linked to the carboxyl groups. Moreover, the connection of the aPD‐L1 was indicated by the disappearance of the characteristic carboxyl, N‐H, and methylene chemical shift signals at δH 4.33 and 3.15, demonstrating successful amidation. In addition, peaks attributed to aPD‐L1 and the TK linker at 2.0–2.5 ppm, 3.1–3.6 ppm were observed, indicating the successful preparation of aPD‐L1 modified by the TK linker.

Finally, PpIX was loaded into the pores of DMSNs‐aPD‐L1 to form DMSNs‐PpIX‐aPD‐L1. UV‐visible spectrophotometer (UV–vis) was used to confirm the loading of PpIX in DMSNs‐PpIX‐aPD‐L1, where the absorption curves changed significantly after loading PpIX (Figure 2C). Furthermore, the characteristic peaks of PpIX and aPD‐L1 were observed in the DMSNs‐PpIX‐aPD‐L1, confirming the loading of PpIX and aPD‐L1 (Figure S4, Supporting Information). Moreover, the loading of PpIX and aPD‐L1 was further confirmed through thermogravimetric analysis as shown in Figure S5, (Supporting Information). These findings suggest the integration of PpIX and aPD‐L1 into DMSNs was effective. Additionally, electron spin resonance (ESR) was used to further investigate whether DMSNs‐PpIX‐aPD‐L1 could generate ^1^O_2_ under US. A strong 1:1:1 signal peak between 3300 and 3400 was found, indicating PpIX was loaded and sensitive to US, producing ^1^O_2_ (Figure 2B). Similarly, oxidation of 1,3‐diphenylbenzofuran (DPBF) was observed when DMSNs‐PpIX‐aPD‐L1 was treated with US, confirming the generation of ^1^O_2_ (Figure S6, Supporting Information).

The surface charge and hydrated particle size of the nanoparticles were then investigated. Figure 2D showed that the negative zeta potential of DMSNs was reversed after NH_2_ modification. Additionally, DMSNs‐PpIX exhibited a reduced potential due to the negative charge of PpIX, confirming successful PpIX loading. The hydrated particle size increased as modification progressed (Figure 2E). Meanwhile, particle stability in various media was monitored for 7 days. As shown in Figure 2F, the particle size of DMSNs‐PpIX‐aPD‐L1 remained stable over the period in three different mediums, indicating good stability and resistance to aggregation.

**Figure 3:**
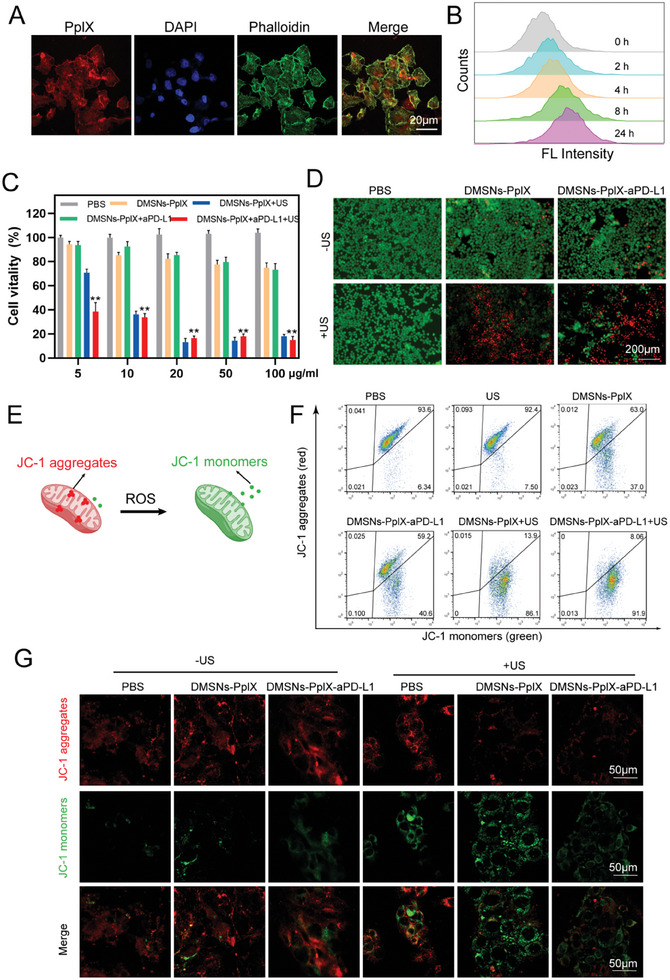
In vitro assessment of anticancer effects of DMSNs‐PpIX‐aPD‐L1. A) CLSM analysis of 4T1 cells after incubation with DMSNs‐PpIX‐aPD‐L1 for 4 h. The cells were counterstained with DAPI (blue, nucleus) and Alexa Fluor 488 phalloidin (green, cytoskeleton). B) Flow cytometric analysis of 4T1 cells postincubation with DMSNs‐PpIX‐aPD‐L1 over various durations. C) The viability of 4T1 cells under different treatment conditions. D) CLSM imagery of 4T1 cells stained with calcein AM/PI following various treatments. E) Illustration of JC‐1 probe analysis for mitochondrial depolarization. F) Determination of JC‐1 probe via flow cytometry with different treatments. G) CLSM imaging of JC‐1 probe in 4T1 cells after different treatments. Quantitative data represent mean values ± SD, (*n* = 3). **p < 0.01.

The in vitro therapeutic efficacy of DMSNs‐PpIX‐aPD‐L1 was investigated next. First, the optimal cellular uptake time was determined by co‐incubating DMSNs‐PpIX‐aPD‐L1 with 4T1 cells and monitoring the process via confocal laser scanning microscopy (CLSM) (**Figure** **3**A). PpIX exhibits distinct red fluorescence co‐localized within 4T1 cells, indicating the uptake of DMSNs‐PpIX‐aPD‐L1 during co‐incubation. Additionally, as incubation time increased, PpIX fluorescence intensity amplified, showing a positive correlation between cellular uptake and time (Figure 3B).

Subsequently, a cell‐counting kit 8 (CCK‐8) assay was conducted to evaluate the intrinsic cellular toxicity of DMSNs‐PpIX‐aPD‐L1 using L929 cell lines. Figure S7 (Supporting Information), indicates that co‐culturing DMSNs‐PpIX‐aPD‐L1 with L929 cells did not exhibit significant biological toxicity over 48 h, confirming the material's biosafety. Next, the therapeutic efficacy of DMSNs‐PpIX‐aPD‐L1 was investigated in 4T1 cells with CCK‐8. Figure 3C showed that DMSNs‐PpIX and DMSNs‐PpIX‐aPD‐L1 were non‐toxic at concentrations below 100 ppm. More importantly, once US was applied, toxicity significantly increased due to the ^1^O_2_ generated by PpIX. This was further corroborated by the cell live/death assay, which showed the most cell death in the DMSNs‐PpIX‐aPD‐L1+US group, indicating that the ^1^O_2_ generated by PpIX induces significant oxidative stress, damaging cancer cells (Figure 3D; Figure S8, Supporting Information). This was visualized using the ROS probe 2′,7′‐dichlorodihydrofluorescein diacetate (DCFH‐DA), where the DMSNs‐PpIX‐aPD‐L1+US group exhibited the most bright fluorescence (Figure S9, Supporting Information). Additionally, since oxidative stress can induce mitochondrial dysfunction, the mitochondrial membrane potential was examined using the 5,5,6,6′‐tetrachloro‐1,1′,3,3′ tetraethylbenzimi‐dazoylcarbocyanine iodide (JC‐1) probe (Figure 3E). Flow cytometry analysis (Figure 3F) revealed that the JC‐1 monomer fraction was 37% and 40.6% when incubated with DMSNs‐PpIX and DMSNs‐PpIX‐aPD‐L1, respectively. Furthermore, this fraction increased to 86.1% and 91.9% when US was applied, indicating significant mitochondrial damage. This was also confirmed by JC‐1 probe fluorescence imaging. As shown in Figure 3G and Figure S10 (Supporting Information), a reduction in red fluorescence and an increase in green fluorescence were observed in the DMSNs‐PpIX+US and DMSNs‐PpIX‐aPD‐L1+US groups, suggesting that the excess ROS generated by US treatment affects mitochondrial membrane potential and induces mitochondrial dysfunction.

Many studies have shown that damaged mitochondria release DAMPs, exposing tumor cells to the immune system, activating anti‐tumor responses, and inducing ICD.^[^ ^37^, ^39^, ^40^, ^41^ ^]^ Therefore, the release of DAMP biomarkers, including ATP, HMGB1, and CRT, was investigated. The DMSNs‐PpIX+US and DMSNs‐PpIX‐aPD‐L1+US groups showed a significant increase in ATP secretion (Figure S11, Supporting Information). Furthermore, cells treated with DMSNs‐PpIX‐aPD‐L1+US exhibited significant HMGB1 release, indicated by a marked reduction in red fluorescence within the cell nuclei, as shown by immunofluorescence staining (Figure S12, Supporting Information). Moreover, in comparison to other treatments, the DMSNs‐PpIX‐aPD‐L1+US treatment enhanced the exposure of CRT on the surface of tumor cells, evidenced by the strongest red fluorescence signal observed (Figure S13, Supporting Information). These findings suggest that under the influence of US, DMSNs‐PpIX‐aPD‐L1 effectively induces ICD by promoting the release of DAMPs from 4T1 tumor cells.

Before in vivo experiments, the potential adverse effects of DMSNs‐PpIX‐aPD‐L1 must be assessed. A hemolytic test was conducted with varying amounts of DMSNs‐PpIX‐aPD‐L1 (Figure S14, Supporting Information). No rupture or lysis of red blood cells were observed, indicating that DMSNs‐PpIX‐aPD‐L1 does not cause adverse reactions in blood. Furthermore, assessments of kidney and liver function markers revealed no negative effects following DMSNs‐PpIX‐aPD‐L1 treatment (Figure S15, Supporting Information), confirming that DMSNs‐PpIX‐aPD‐L1 did not impact the host's biosafety.

The accumulation of DMSNs‐PpIX‐aPD‐L1 in the tumor was assessed by tracking its distribution after intravenous injection. The fluorescence intensity of DMSNs‐PpIX‐aPD‐L1 gradually increased, with the strongest signal detected 24 h post‐injection (Figure S16, Supporting Information). Additionally, the fluorescence signal in the tumor was significantly higher than in other organs, indicating that most DMSNs‐PpIX‐aPD‐L1 accumulated in the tumor. The strong signal in the liver was attributed to DMSNs‐PpIX‐aPD‐L1 presence in the liver's reticuloendothelial system.

**Figure 4:**
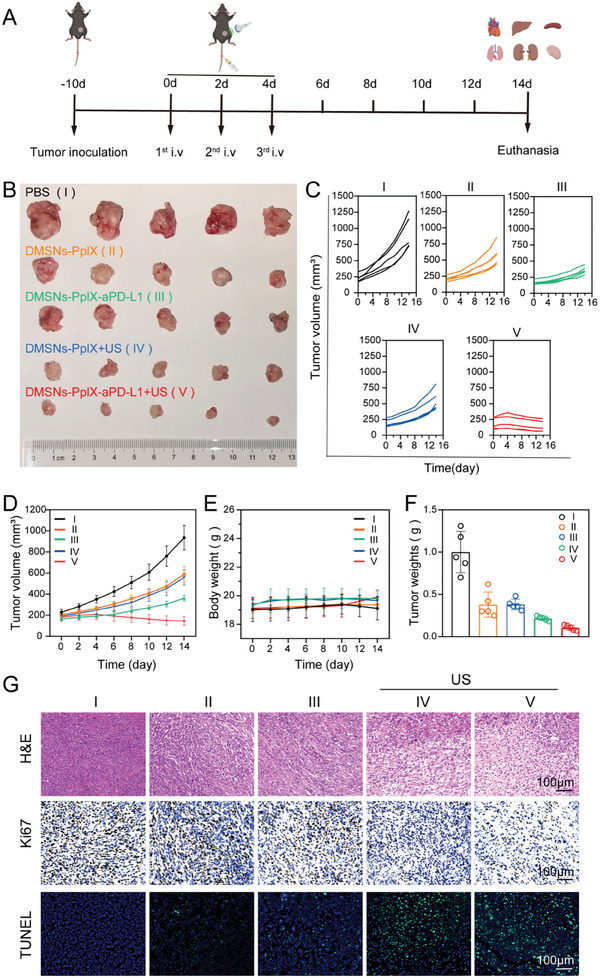
In vivo evaluation of anticancer efficacy and safety of DMSNs‐PpIX‐aPD‐L1. In vivo anticancer efficacy was evaluated as follows: A) A schematic representation of the therapeutic procedure in vivo. B) Images of 4T1 tumors excised on day 14 post various treatments. C) Growth progression of individual tumors. D) Average tumor volume. E) Average body weight. F) Average tumor weight after different treatments. G) H&E, Ki67, and TUNEL staining analyses of tumor samples from each treatment group at the 14‐day mark. Quantitative data represent mean values ± SD, (*n* = 3).

Driven by favorable biocompatibility, efficient tumor uptake, and strong in vitro anticancer efficacy, the therapeutic effect of DMSNs‐PpIX‐aPD‐L1 was further assessed in a 4T1‐bearing mouse model. The mice were randomly divided into five groups: (I) control, (II) DMSNs‐PpIX, (III) DMSNs‐PpIX+US, (IV) DMSNs‐PpIX‐aPD‐L1, and (V) DMSNs‐PpIX‐aPD‐L1+US. PBS, DMSNs‐PpIX, and DMSNs‐PpIX‐aPD‐L1 were injected via tail vein on days 0, 2, and 4, and US treatments were administered 24 h after each injection (**Figure** **4**A). After 14 days of treatment, tumors were collected (Figure 4B). Tumor volumes for each group were recorded every 2 days (Figure 4C,D). Additionally, body weight changes in treated mice were recorded throughout the treatment (Figure 4E). No significant changes were observed, indicating the treatment did not cause notable side effects on the mice's health. Additionally, aPD‐L1 monotherapy was performed, and tumor volume and weight were recorded in Figure S17 (Supporting Information). However, due to limited therapeutic outcomes, 7 cycles of aPD‐L1 treatment were conducted. Comparing the results from Figure 4F and Figure S17 (Supporting Information), DMSNs‐PpIX‐aPD‐L1+US showed the most significant tumor inhibition

The therapeutic effects of different treatment on tumor were further assessed using hematoxylin and eosin (H&E) and Ki67 staining, as well as transferase dUTP Nick‐End Labeling (TUNEL) assay (Figure 4G). The H&E and Ki67 staining showed that DMSNs‐PpIX‐aPD‐L1+US induced significant apoptosis, as evidenced by altered cell morphology, nuclear condensation, and inhibition of proliferation. The TUNEL assay also indicated that the highest percentage of tumor cells were killed in the DMSNs‐PpIX‐aPD‐L1+US group.

**Figure 5:**
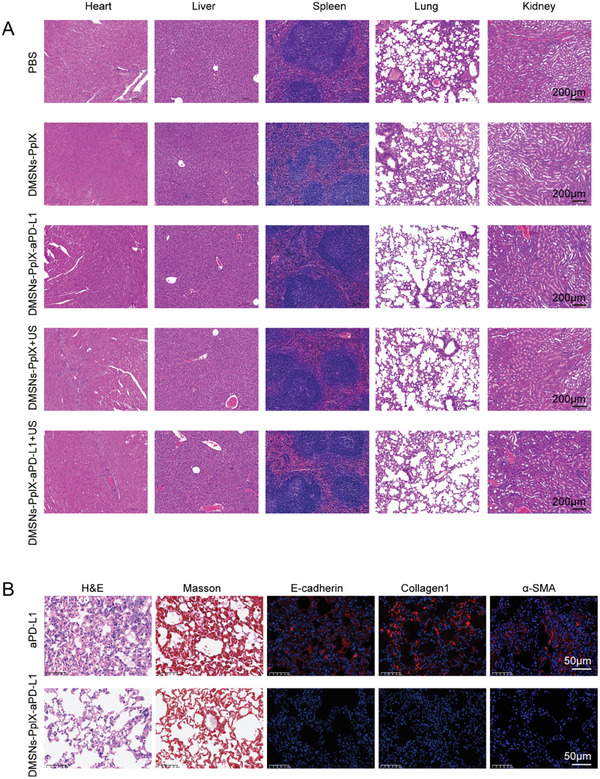
Histopathological analysis of tissues and immunomodulatory effects. A) H&E staining of the slices of heart, liver, spleen, lung, and kidney harvested from tumor‐bearing mice after different treatments for 14 days. B) Histological analysis of mouse lung tissue sections after 7 cycles of treatments, including H&E staining, Masson's trichrome staining (red myofiber, blue collagenous fiber), and immunohistochemical staining for E‐cadherin, collagen I, and α‐SMA.

After treatments, key organs were harvested and examined histologically using H&E staining. The results showed no apparent pathological toxicity during the treatment period (**Figure** **5**A), indicating that DMSNs‐PpIX‐aPD‐L1 treatments caused no significant histological abnormalities. In contrast, H&E staining of lung tissues after aPD‐L1 monotherapy showed significant pulmonary fibrosis due to irAEs (Figure 5B). To further evaluate the benefits of DMSNs‐PpIX‐aPD‐L1 in mitigating irAEs, mice underwent 7 cycles of DMSNs‐PpIX‐aPD‐L1 treatment for comparison. Lung tissues from both treatments were then collected for H&E, Masson's trichrome, and immunohistochemical staining. As shown in Figure 5B, after 7 cycles of DMSNs‐PpIX‐aPD‐L1 treatment, minimal pulmonary damage and fibrosis were observed, while the aPD‐L1 monotherapy group showed significant pulmonary fibrosis. Additionally, immunohistochemical staining of E‐cadherin, collagen I, and α‐SMA indicated that DMSNs‐PpIX‐aPD‐L1 treatment led to fewer fibers and less collagen deposition. The release of aPD‐L1 was triggered by US in DMSNs‐PpIX‐aPD‐L1, resulting in on‐demand release in the tumor and reduced adverse immune responses in other organs compared to aPD‐L1 monotherapy.

**Figure 6:**
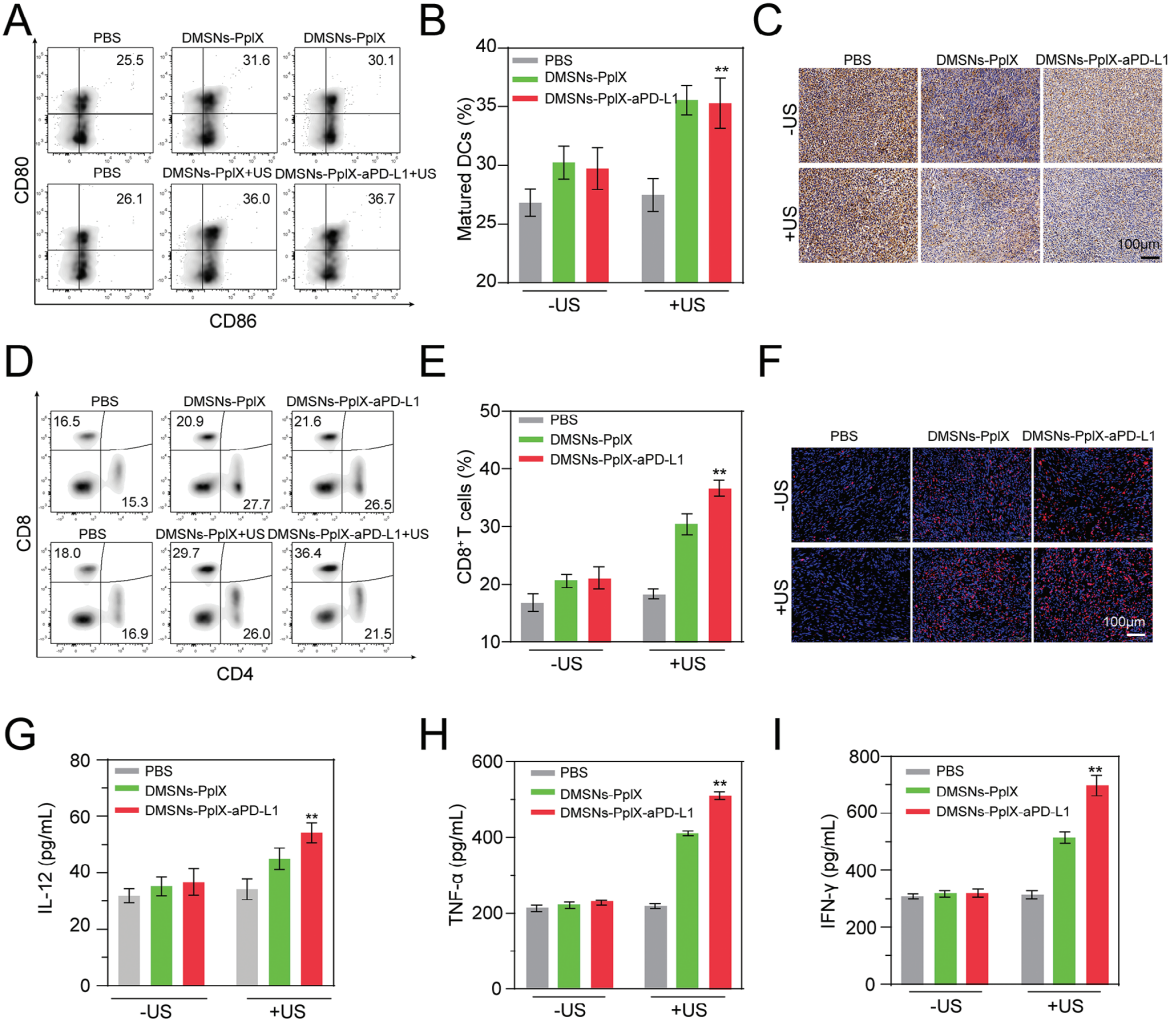
Immune response activation by DMSNs‐PpIX‐aPD‐L1. A) Assessment of mature DCs in the tumor‐draining lymph nodes of treated mice over 3 days using flow cytometry. B) Quantification of mature DCs percentages in tumor‐draining lymph nodes after various treatments. C) Immunohistochemistry of PD‐L1 expression post treatment. D) Evaluation of CD4^+^ T cells and CD8^+^ T cells within the tumor 10 days after different treatments. E) Enumeration of CD8^+^ T cell populations. F) Immunofluorescent detection of CD8^+^ T cells in treated groups. Cytokine assessments after 3 days of treatment: G) IL‐12, H) TNF‐α, and I) IFN‐γ. Data are expressed as mean ± SD (*n* = 3); **p < 0.01.

Building on earlier findings that DMSNs‐PpIX‐aPD‐L1 combined with US can trigger ICD through the effective release of DAMPs, we conducted an analysis of immunofluorescence staining images of the tumor. In the DMSNs‐PpIX‐aPD‐L1+US treatment group, there was a noticeable increase in fluorescence intensity for both HMGB1 (Figure S18, Supporting Information) and CRT (Figure S19, Supporting Information). These observations prompted us to further explore the potential immunological outcomes of our treatment strategy, focusing on enhancing DCs maturation and T cell infiltration, as both ICD and PD‐L1 inhibition in established tumors can lead to effective tumor suppression by promoting DCs maturation and T cell reactivation.^[^ ^42^, ^43^, ^44^ ^]^ **Figure** **6**A,B showed that DCs maturation significantly increased in the DMSNs‐PpIX‐aPD‐L1+US group (≈36%), much higher than in the control group (25.5%), indicating a stronger immune response. Immunohistochemical analysis of tumor section showed a reduction in PD‐L1 expression after DMSNs‐PpIX‐aPD‐L1+US treatment (Figure 6C), indicating effective suppression of PD‐L1 on tumor cells.

CTLs (CD8^+^ T cells) and helper T cells (CD4^+^ T cells) infiltrating tumors are crucial for initiating immune responses against cancer. Therefore, the presence of CD8^+^ and CD4^+^ T cells in tumors was examined after different treatments (Figure 6D,E; Figure S20, supporting information). Consistent with DCs maturation, a higher CD8^+^ level was seen in the DMSNs‐PpIX‐aPD‐L1+US group (36.4%) compared to the control group (16.5%), indicating stronger immune activation from the synergistic effect of DMSNs‐PpIX‐aPD‐L1 and US. Immunofluorescence analysis further confirmed increased CD8^+^ T cell infiltration in the tumor after DMSNs‐PpIX‐aPD‐L1+US treatment (Figure 6F). Additionally, intratumoral inflammatory cytokine levels are linked to immunity, thus interleukin‐12 (IL‐12), tumor necrosis factor‐α (TNF‐α), and interferon‐γ (IFN‐γ) were assessed (Figure 6G–I). DMSNs‐PpIX‐aPD‐L1+US treatment significantly promoted the secretion of these cytokines in tumors. These results suggest that DMSNs‐PpIX‐aPD‐L1+US not only inhibited established tumor growth but also activated immune responses, offering defensive effects to suppress tumor recurrence.

**Figure 7:**
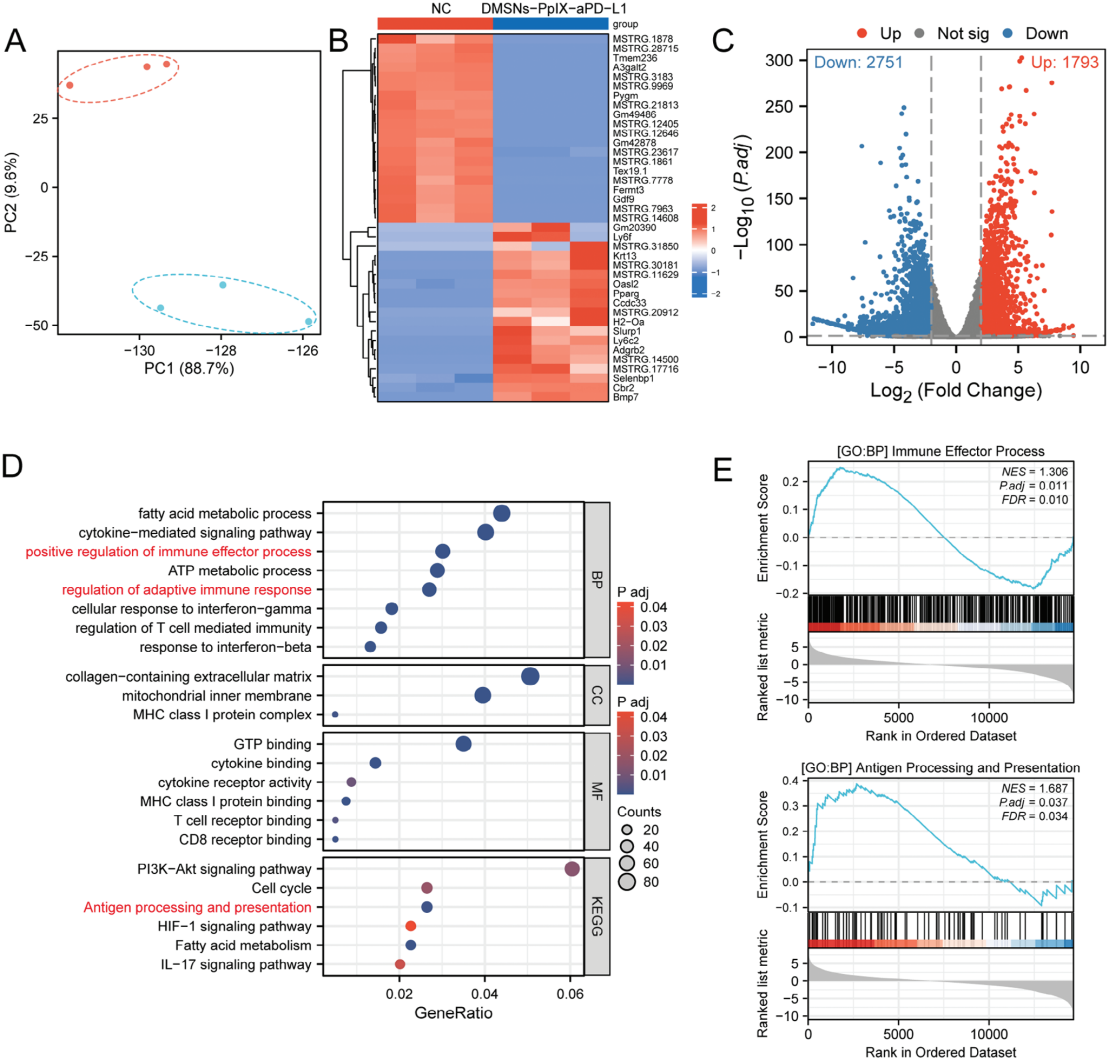
In vivo immune‐related gene expression analysis: A) PCA plot, B) heat map, and C) volcano plot comparing the PBS group and DMSNs‐PplX‐aPD‐L1 treated group. D) Pathways enriched by GO analysis and KEGG analysis of genes elevated in the DMSNs‐PplX‐aPD‐L1 treatment group. E) GSEA data of pathways enriched in the DMSNs‐PplX‐aPD‐L1 treatment group.

To explore the mechanism of immune activation at the gene level, we conducted transcriptomic analysis of DMSNs‐PpIX‐aPD‐L1+US and PBS‐treated mice bearing 4T1 cells. Principal component analysis (PCA) revealed substantial variations in genomic expression levels after treatment with DMSNs‐PpIX‐aPD‐L1+US and PBS (**Figure** **7**A). Whole‐genome analysis showed that compared to the PBS group, the DMSNs‐PpIX‐aPD‐L1+US group had 1793 up‐regulated and 2751 down‐regulated genes. The most significant differentially expressed genes are shown in the heat map (Figure 7B,C). Gene Ontology (GO) and Kyoto Encyclopedia of Genes (KEGG) pathway analyses were performed to reveal biological information, including regulatory pathways and biomolecular functions. GO analysis showed that the most enriched pathways involved fatty acid metabolism, inflammatory cytokine signaling, positive immune regulation, and adaptive immune response regulation, all related to immune activation. KEGG analysis further showed that multiple immune‐related pathways, such as antigen presentation and IL‐17 signaling, were enriched in DMSNs‐PpIX‐aPD‐L1+US‐treated mice (Figure 7D). Gene Set Enrichment Analysis (GSEA) also confirmed these results, with immune processes and antigen presentation enriched in DMSNs‐PpIX‐aPD‐L1+US‐treated mice (Figure 7E). Moreover, we used GO analysis combined with fold change (FC) to create a chord diagram. The results showed activation of pathways including ATP metabolic process, regulation of mitochondrial membrane potential, and antigen processing and presentation via MHC class Ib (Figure S21, Supporting Information). Our previous experiments also demonstrated a significant increase in ATP release, suggesting that DMSNs‐PpIX‐aPD‐L1+US treatment may induce mitochondrial damage leading to ATP release, thereby activating downstream immune responses. ATP release, as a signaling molecule, promotes the recruitment and activation of immune cells within the tumor microenvironment, providing a crucial driving force for anti‐tumor immunity. Additionally, we generated a heatmap based on the expression of genes related to the ATP metabolic process, which indicated that DMSNs‐PpIX‐aPD‐L1+US could enhance the tumor immune response (Figure S22, Supporting Information). Therefore, these results showed that DMSNs‐PpIX‐aPD‐L1 exhibited effectively inhibits tumors and elicited immune responses in mice through combined SDT and aPD‐L1 therapy. These findings offer valuable insights for designing US‐activated sono‐immunotherapy platforms.

## 3 Conclusion

A novel US‐activated sono‐immnotherapy nanodrug, DMSNs‐PpIX‐aPD‐L1, was successfully developed, providing precise breast cancer treatment while reducing aPD‐L1 immunotoxicity. Leveraging the non‐invasive deep tissue penetration of US, DMSNs‐PpIX‐aPD‐L1 can be directly activated within the tumor. This activation releases ^1^O_2_, breaking down the TK linker and releasing aPD‐L1 on demand, thus initiating a sequenced spatiotemporal breast cancer immunotherapy. Additionally, this precise treatment boosts the immune response within the tumor while significantly reducing pulmonary fibrosis commonly seen in aPD‐L1 treatments. This approach ensures maximal treatment efficiency with minimal side effects, which is crucial for breast cancer immunotherapy in preventing pulmonary fibrosis.

## 4 Experimental Section

### Materials

PpIX was obtained from China National Pharmaceutical Group Chemical Reagent Co., Ltd. Mouse PD‐L1 inhibitor (Anti‐Mouse PD‐L1/B7‐H1 Antibody (10F.9G2)), JC‐1 probe, and Masson's Trichrome Stain Kit were procured from Thermo Scientific. The mouse 4T1 cell line was provided by the Cell Bank of the Chinese Academy of Sciences. Flow cytometry antibodies including FITC‐CD3, APC‐CD4, PE‐CD8, PE‐CD11c, FITC‐CD86, and APC‐CD80 were supplied by BioLegend (USA). IL‐12 kit, TNF‐α Kit, and IFN‐γ Kit were obtained from Elabscience. The immunohistochemistry Kit was sourced from Shanghai ExCell Bio.

### Synthesis of DMSNs‐NH_2_

Cetyltrimethylammonium bromide (1.535 g), NaCl (0.672 g), and triethanolamine (0.347 g) were dissolved in water (100 mL) and stirred. Then tetraethyl orthosilicate (10 mL) was added and stirred to obtain DMSNs. Then, DMSNs (160 mg) was dissolved in ethanol (100 mL), and (3‐aminopropyl) trimethoxysilane (120 µL) was added to react for 4 h. DMSNs‐NH_2_ was obtained after centrifugation.

### Synthesis of DMSNs‐PpIX

PpIX (20 mg) was dissolved in methanol (3 mL), followed by adding to DMSNs‐NH_2_ (88 mg in 20 mL of water) and reacted for 24 h to obtain DMSNs‐PpIX.

### Synthesis of DMSNs‐PpIX‐aPD‐L1

TK linker (34 mg) was dissolved in water, EDC (139.2 mg) and NHS (77.6 mg) were added and reacted for 15 min. aPD‐L1 (17.6 mg) was added and further reacted for 12 h. The solution was dropwisely added to DMSNs‐PpIX (88 mg in 20 mL of water) and reacted for 24 h to obtain DMSNs‐PpIX‐aPD‐L1.

### Characterization

Images of the morphology were captured using an aberration‐corrected transmission electron microscope. The Zeta potential and dynamic light scattering of different samples were measured using a Malvern Zetasizer Nano ZS90, while their UV–vis absorption spectra were captured with a UV‐1900 spectrophotometer. The Bruker EMX1598 spectrometer was used to acquire ESR spectra of the samples.

### ESR Measurement of ^1^O_2_

For detecting ^1^O_2_ produced by DMSNs‐PpIX‐aPD‐L1 under US radiation, TEMP was utilized to trap ^1^O_2_. The duration of US radiation was 5 min (1 W cm^−2^,1.0 MHz).

### In Vitro Cytotoxicity

4T1 cells were cultured with DMSNs‐PpIX‐aPD‐L1 at varying concentrations (0, 5, 10, 20, 50, and 100 µg mL^−1^) for 24 h. The medium was then replaced with CCK‐8 solution and incubated for 1 h.

### Mitochondrial Membrane Potential Analysis

4T1 cells cultured with DMSNs‐PpIX and DMSNs‐PpIX‐aPD‐L1 for 4 h. US treatments were then conducted and incubated for an additional 20 h. Afterward, JC‐1 were then added to incubate for 20 min. The cells were analyzed via flow cytometry. Untreated 4T1 cells served as the control group.

### Tumor Model

The animal experiments involved the use of C57BL/6 mice, which were obtained from the Zhejiang Institute of Biological Products. All procedures involving mice were conducted in accordance with the guidelines approved by the Experimental Animal Welfare Ethics Committee of Nanjing Medical University (NO. 2 310 081).

### In Vivo Biocompatibility of DMSNs‐PpIX‐aPD‐L1

Healthy C57BL/6 mice were intravenously administered varying doses of DMSNs‐PpIX‐aPD‐L1. Blood samples were collected on days 3, 7, and 30 post‐injection for hemolysis assay and biochemical assays. Major organs, including the heart, liver, spleen, lungs, and kidneys, were collected for histological examination using H&E staining.

### Tumor Suppression Experiments In Vivo

4T1 cells suspended in PBS were subcutaneously inoculated into the upper legs of C57BL/6 mice. Once tumor volumes were ≈100 mm^3^, the mice were randomly divided into five groups, each containing five mice: 1) Control, 2) DMSNs‐PpIX, 3) DMSNs‐PpIX‐aPD‐L1, 4) DMSNs‐PpIX+US, and 5) DMSNs‐PpIX‐aPD‐L1+US. The mice received intravenous administration of the respective materials. In the US groups, tumors received US treatment (1.0 MHz, 1 W cm^−2^, 5 min) 24 h after intravenous injection. Throughout the treatment period, both tumor size and body weight were measured every two days. The aPD‐L1 monotherapy group was treated similarly, except the mice were treated with 7 cycles over 14 days and euthanized on day 15.

### H&E, Ki67, and TUNEL Staining

For histological analysis, tumors were excised from mice in each group 48 h following the various treatments. These tumors were fixed in 10% paraformaldehyde, embedded in paraffin, sliced into ≈4 µm sections, and stained with H&E, Ki67, and TUNEL staining was conducted.

### Maturation of DCs and Analysis of CD4^+^ and CD8^+^ T Cells

To evaluate the maturation of DCs marked by CD11c^+^CD80^+^CD86^+^ expression, lymphocytes were harvested from mouse lymph node tissue and stained with different antibodies. For the examination of infiltrating lymphocytes, cells were isolated from tumor tissues, and PBS containing 1 mg mL^−1^ collagenase, 1 mg mL^−1^ hyaluronidase, and 10 µg mL^−1^ DNaseI was used to prepare single‐cell suspensions by incubation for 2 h at 37 ^°^C. Analysis of CD8^+^ T cells and CD4^+^ T cells was performed using specific antibodies via flow cytometry.

### mRNA Sequencing Analysis

mRNA was extracted from 4T1 cells treated with PBS and DMSNs‐PpIX‐aPD‐L1 using Trizol. The extracted mRNA was quantified using a NanoDrop 2000 spectrophotometer (Thermo, USA), and its integrity was assessed with an Agilent 2100 bioanalyzer (Suzhou, China). Sequencing libraries were prepared when the total mRNA concentration exceeded 50 ng µL^−1^, with an optical density range of 1.8–2.2 and an RNA integrity number of 7 or above. Gene expression was quantified as fragments per kilobase of exon model per million mapped fragments. GO terms and KEGG pathways associated with the differentially expressed genes were identified using GO‐Term Finder and the Annotation, Visualization, and Integrated Discovery Database to explore their functions.

### Statistical Analysis

The statistical analysis of the data was conducted using GraphPad Prism (version 8.0, GraphPad Software), with quantification performed using ImageJ. The data were presented as mean ± standard error of the mean. To evaluate the effects of multiple treatments, one‐way analysis of variance followed by Tukey's posthoc test was utilized, with a statistical significance level of *p* < 0.05 considered significant. *p < 0.05, **p < 0.01, ***p < 0.001, and ****p < 0.0001.

## Conflict of Interest

The authors declare no conflict of interest.

## Supporting information



Supporting Information

## Data Availability

Research data are not shared.

## References

[1] N. Harbeck , F. Penault‐Llorca , J. Cortes , M. Gnant , N. Houssami , P. Poortmans , K. Ruddy , J. Tsang , F. Cardoso , Nat. Rev. Dis. Primers 2019, 5, 66. 31548545 10.1038/s41572-019-0111-2

[2] S. Loibl , P. Poortmans , M. Morrow , C. Denkert , G. Curigliano , Lancet 2021, 397, 1750. 33812473 10.1016/S0140-6736(20)32381-3

[3] R. Siegel , A. Giaquinto , A. Jemal , CA Cancer J. Clin. 2024, 74, 12. 38230766 10.3322/caac.21820

[4] R. Fitzgerald , A. Antoniou , L. Fruk , N. Rosenfeld , Nat. Med. 2022, 28, 666. 35440720 10.1038/s41591-022-01746-x

[5] T. Fan , M. Zhang , J. Yang , Z. Zhu , W. Cao , C. Dong , Signal Transduction Targeted Ther. 2023, 8, 450. 10.1038/s41392-023-01674-3 PMC10716479 38086815

[6] Y. Lu , Y. Gao , H. Yang , Y. Hu , X. Li , Mil. Med. Res 2022, 9, 69. 36503490 10.1186/s40779-022-00433-9 PMC9743634

[7] D. Wang , X. Wu , Y. Sun , Signal Transduction Targeted Ther. 2022, 7, 331. 10.1038/s41392-022-01136-2 PMC9485144 36123348

[8] Y. Zhang , Z. Zhang , Cell. Mol. Immunol. 2020, 17, 807. 32612154 10.1038/s41423-020-0488-6 PMC7395159

[9] A. Waldman , J. Fritz , M. Lenardo , Nat. Rev. Immunol. 2020, 20, 651. 32433532 10.1038/s41577-020-0306-5 PMC7238960

[10] G. Bantug , C. Hess , Nat. Immunol. 2023, 24, 2008. 38012409 10.1038/s41590-023-01675-y

[11] M. Li , C. Lin , A. Shen , X. Ma , J. Ni , J. Wu , W. Wang , P. Wang , X. Gao , Adv. Funct. Mater. 2024, 34, 2315171.

[12] C. Shi , Q. Zhang , Y. Yao , F. Zeng , C. Du , S. Nijiati , X. Wen , X. Zhang , H. Yang , H. Chen , Z. Guo , X. Zhang , J. Gao , W. Guo , X. Chen , Z. Zhou , Nat. Nanotechnol. 2023, 18, 86. 36536041 10.1038/s41565-022-01261-7

[13] X. Zhao , K. Zhang , Y. Wang , W. Jiang , H. Cheng , Q. Wang , T. Xiang , Z. Zhang , J. Liu , J. Shi , Adv. Funct. Mater. 2022, 32, 2108883.

[14] Y. Murciano‐Goroff , A. Warner , J. Wolchok , Cell Res. 2020, 30, 507. 32467593 10.1038/s41422-020-0337-2 PMC7264181

[15] M. Binnewies , E. Roberts , K. Kersten , V. Chan , D. Fearon , M. Merad , L. Coussens , D. Gabrilovich , S. Ostrand‐Rosenberg , C. Hedrick , R. Vonderheide , M. Pittet , R. Jain , W. Zou , T. Howcroft , E. Woodhouse , R. Weinberg , M. Krummel , Nat. Med. 2018, 24, 541. 29686425 10.1038/s41591-018-0014-x PMC5998822

[16] W. Nie , W. Wei , L. Zuo , C. Lv , F. Zhang , G. Lu , F. Li , G. Wu , L. Huang , X. Xi , H. Xie , ACS Nano 2019, 13, 1469. 30763076 10.1021/acsnano.8b07141

[17] B. Yang , J. Gao , Q. Pei , H. Xu , H. Yu , Adv. Sci. 2020, 7, 2002365. 10.1002/advs.202002365 PMC7709995 33304763

[18] K. You , Q. Wang , M. Osman , D. Kim , Q. Li , C. Feng , L. Wang , K. Yang , BMEMat. 2024, 2, 12067.

[19] X. Zhu , S. Chen , X. Hu , L. Zhao , Y. Wang , J. Huang , J. Chen , Y. Qiu , X. Zhang , M. Wang , X. Yang , Y. Zhang , Y. Zhu , Adv. Mater. 2023, 35, 2207198. 10.1002/adma.202207198 36314411

[20] Y. Zhao , Y. Xie , S. Van Herck , S. Nassiri , M. Gao , Y. Guo , L. Tang , Sci. Adv. 2021, 7, abg7291. 10.1126/sciadv.abg7291 PMC8442900 34516776

[21] X. Yan , K. Li , T. Xie , X. Jin , C. Zhang , Q. Li , J. Feng , C. Liu , X. Zhang , Angew. Chem., Int. Ed. Engl. 2024, 63, 202318539. 10.1002/anie.202318539 38303647

[22] W. Wang , W. Xu , J. Zhang , Y. Xu , J. Shen , N. Zhou , Y. Li , M. Zhang , B. Tang , ACS Nano 2024, 18, 4089. 38270107 10.1021/acsnano.3c08054

[23] W. Wang , Y. Gao , J. Xu , T. Zou , B. Yang , S. Hu , X. Cheng , Y. Xia , Q. Zheng , Adv. Sci. 2024, 11, 2307143. 10.1002/advs.202307143 PMC11005733 38308097

[24] D. Li , K. Zhang , K. Wang , R. Peng , X. Liu , Y. Miao , Y. Lan , R. Wang , L. Dong , Y. Luo , Nano Lett. 2024, 24, 8996. 38995813 10.1021/acs.nanolett.4c02027

[25] G. Hu , J. Wang , L. Chen , H. Zheng , Y. Lan , K. Wang , X. Liu , H. You , Y. Luo , L. Dong , Adv. Funct. Mater. 2024, 34, 2401897.

[26] W. Tang , J. Wu , L. Wang , K. Wei , Z. Pei , F. Gong , L. Chen , Z. Han , Y. Yang , Y. Dai , X. Cui , L. Cheng , ACS Nano 2024, 18, 10495. 38556991 10.1021/acsnano.3c11818

[27] Y. Xu , D. Tang , L. Li , X. Li , Q. Chang , H. Xiao , W. Li , Adv. Funct. Mater. 2024, 34, 2315385.

[28] Y. Li , W. Chen , Y. Kang , X. Zhen , Z. Zhou , C. Liu , S. Chen , X. Huang , H. Liu , S. Koo , N. Kong , X. Ji , T. Xie , W. Tao , Nat. Commun. 2023, 14, 6973. 37914681 10.1038/s41467-023-42509-7 PMC10620173

[29] C. Zhang , K. Pu , Adv. Mater. 2023, 35, 2303059. 10.1002/adma.202303059 37263297

[30] Y. Zhang , N. Pang , X. Huang , W. Meng , L. Meng , B. Zhang , Z. Jiang , J. Zhang , Z. Yi , Z. Luo , Z. Wang , L. Niu , Fundam. Res. 2023, 3, 469. 38933758 10.1016/j.fmre.2022.02.010 PMC11197585

[31] M. Zhang , L. Dong , D. Li , L. Zhu , R. Peng , X. Liu , K. Wang , X. Wang , Y. Zhu , H. Sun , Y. Luo , Adv. Funct. Mater. 2023, 33, 2303451.

[32] B. Xu , S. Li , R. Shi , H. Liu , Signal Transduction Targeted Ther. 2023, 8, 435. 10.1038/s41392-023-01654-7 PMC10667354 37996406

[33] P. Dogra , N. Adolphi , Z. Wang , Y. Lin , K. Butler , P. Durfee , J. Croissant , A. Noureddine , E. Coker , E. Bearer , V. Cristini , C. Brinker , Nat. Commun. 2018, 9, 4551. 30382084 10.1038/s41467-018-06730-z PMC6208419

[34] R. Kankala , Y. Han , J. Na , C. Lee , Z. Sun , S. Wang , T. Kimura , Y. Ok , Y. Yamauchi , A. Chen , K. Wu , Adv. Mater. 2020, 32, 1907035. 10.1002/adma.201907035 32319133

[35] N. Yang , H. Li , C. Cao , L. Zhao , X. Song , W. Wang , W. Xu , Y. Zhang , P. Chen , X. Dong , Fundam. Res. 2024, 4, 178. 38933846 10.1016/j.fmre.2022.04.021 PMC11197737

[36] C. Liang , J. Xie , S. Luo , C. Huang , Q. Zhang , H. Huang , P. Zhang , Nat. Commun. 2021, 12, 5001. 34408151 10.1038/s41467-021-25303-1 PMC8373944

[37] S. Li , X. Chen , S. Guan , Z. Wang , W. Cao , G. Luo , X. Ling , ACS Nano 2023, 17, 15165. 37490051 10.1021/acsnano.3c04785

[38] N. Yang , X. Sun , Y. Zhou , X. Yang , J. You , Z. Yu , J. Ge , F. Gong , Z. Xiao , Y. Jin , Z. Liu , L. Cheng , Sci. Bull. 2023, 68, 1772. 10.1016/j.scib.2023.07.025 37516662

[39] Y. Wang , J. Wang , S. Tao , Z. Liang , R. Xie , N. Liu , R. Deng , Y. Zhang , D. Deng , G. Jiang , Diabetes Metab. Res. Rev. 2024, 40, 3733. 10.1002/dmrr.3733 37823338

[40] M. Lin , N. Liu , Z. Qin , Y. Wang , Acta Pharmacol. Sin 2022, 43, 2439. 35233090 10.1038/s41401-022-00879-6 PMC9525705

[41] Y. Huang , W. Jiang , R. Zhou , Nat. Rev. Immunol. 2024, 24, 703. 38684933 10.1038/s41577-024-01027-3

[42] M. Xiong , Y. Zhang , H. Zhang , Q. Shao , Q. Hu , J. Ma , Y. Wan , L. Guo , X. Wan , H. Sun , Z. Yuan , H. Wan , Adv. Sci. 2024, 11, 2402465. 10.1002/advs.202402465 PMC11267356 38728587

[43] H. Wang , Z. He , Y. Gao , D. Feng , X. Wei , Y. Huang , J. Hou , S. Li , W. Zhang , Adv. Sci. 2023, 10, 2302422. 10.1002/advs.202302422 PMC10558672 37544896

[44] S. Oh , D. Wu , J. Cheung , A. Navarro , H. Xiong , R. Cubas , K. Totpal , H. Chiu , Y. Wu , L. Comps‐Agrar , A. Leader , M. Merad , M. Roose‐Germa , S. Warming , M. Yan , J. Kim , S. Rutz , I. Mellman , Nat. Cancer 2020, 1, 681. 35122038 10.1038/s43018-020-0075-x

