# Supplementary material for: Ultrasound‐Activated Precise Sono‐Immunotherapy for Breast Cancer with Reduced Pulmonary Fibrosis
Source: Adv Sci (Weinh). 2024 Dec 16;12(5):2407609. doi: 10.1002/advs.202407609 (PMC11791983; doi:10.1002/advs.202407609)
Supplement: Supplementary file 1 — Supporting Information [file ADVS-12-2407609-s001.docx]

**Ultrasound-activated Precise Sono-immunotherapy for Breast Cancer and Alleviation of Pulmonary Fibrosis**

*Xiang Li^#^*, Gao He^#^, Hui Jin^#^, Xinyu Xiang, Dong Li, Renmiao Peng, Jing Tao, Xinping Li, Kaiyang Wang*, Yu Luo*, Xiaoan Liu**

*X. Li, J. Tao, XinPing. Li*

Department of Thyroid-Breast Surgery, The Fourth Affiliated Hospital of Nanjing Medical University, 298 Nanpu Road, Nanjing, 210032, Jiangsu Province, P.R.China.

Email: xiangli@njmu.edu.cn

*X. Liu*

Breast Disease Center, The First Affiliated Hospital of Nanjing Medical University, 300 Guangzhou Road,Nanjing, 210029, Jiangsu Province, P. R. China.

Email: liuxiaoan@jsph.org.cn

*G. He,*

Breast Disease Center, The First Affiliated Hospital of Nanjing Medical University, 300 Guangzhou Road,Nanjing, 210029, Jiangsu Province, P. R. China.

The Afffliated Taizhou People’s Hospital of Nanjing Medical University, Taizhou School of Clinical Medicine, Nanjing Medical University, 366 Taihu Road, Taizhou, Jiangsu 225300, PR China

*H.Jin*

Department of Breast surgery, The Affiliated Tumor Hospital of Nantong University ,30 tongyang north road,Nantong, 226361, Jiangsu Province , P. R. China.

*X. Xiang, D. Li, R. Peng, K. Wang, Y. Luo*

Shanghai Engineering Research Center of Pharmaceutical Intelligent Equipment, Shanghai Frontiers Science Research Center for Druggability of Cardiovascular Non-coding RNA, Institute for Frontier Medical Technology School of Chemistry and Chemical Engineering Shanghai University of Engineering Science, Shanghai, 201620, P. R. China

E-mail: kaiyang.wang@sues.edu.cn; yuluo@sues.edu.cn


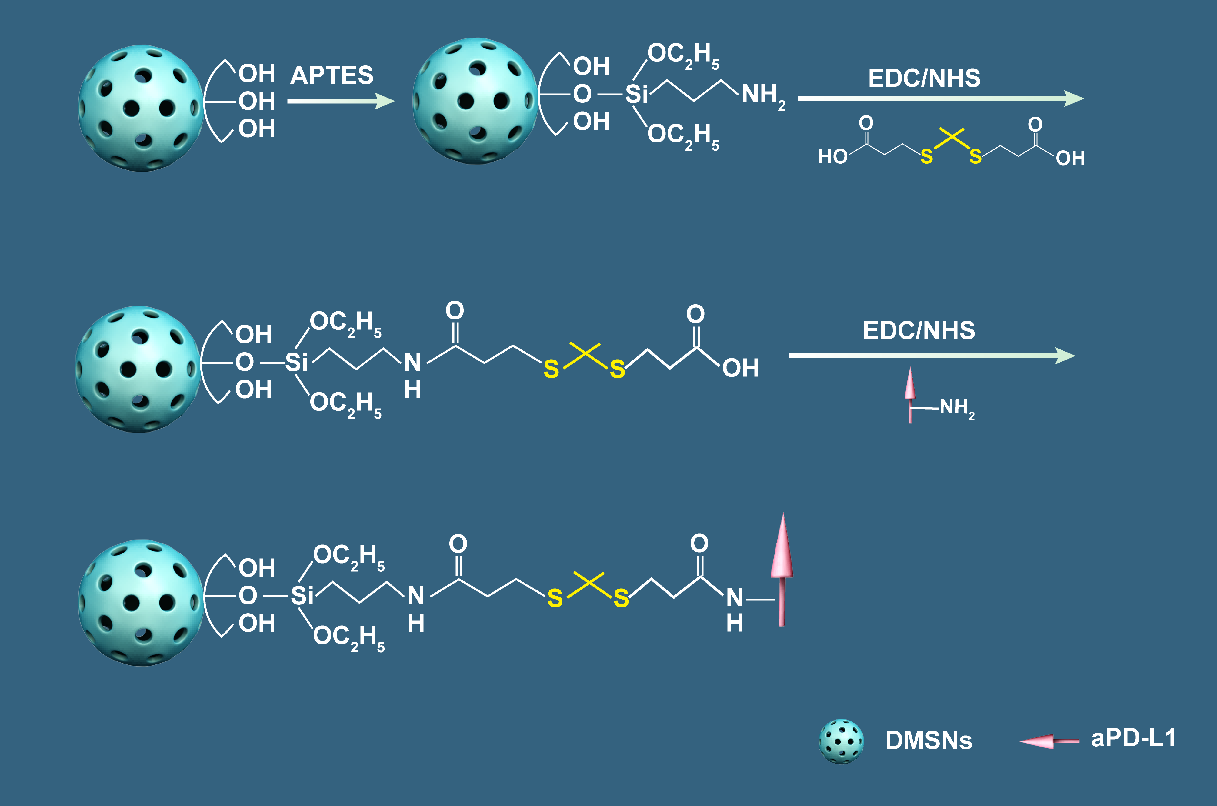


Figure S1. The pathway of conjugating aPD-L1 onto DMSNs


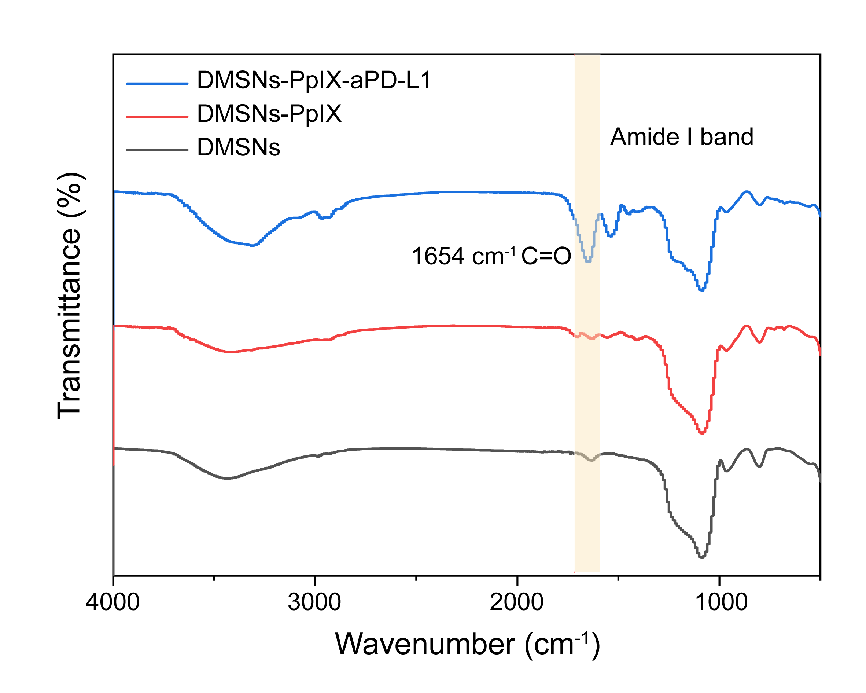


Figure S2. FT-IR analysis of aPD-L1 antibody loading. FT-IR bands ranging from ~1600 to 1700 cm^-1^ were attributed to the amide l band of the protein.


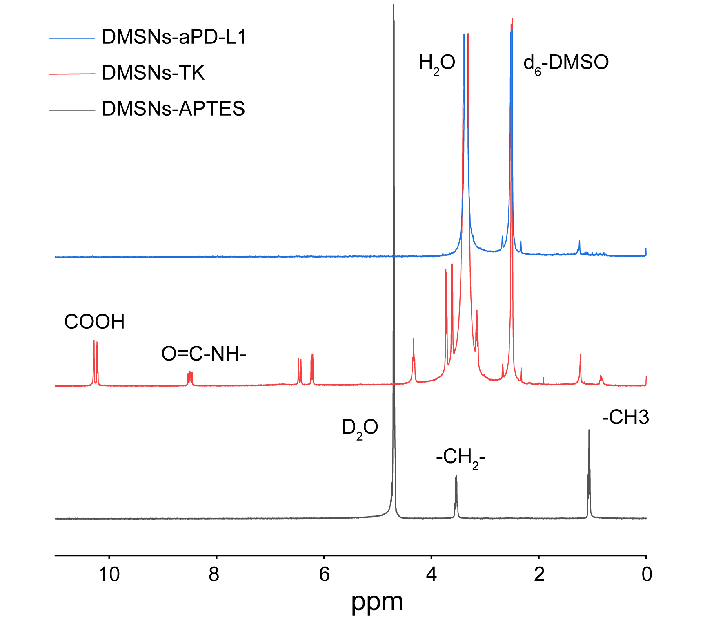


Figure S3. ^1^H NMR spectra of DMSNs-APTES, DMSNs-TK, and DMSNs-aPD-L1, respectively.


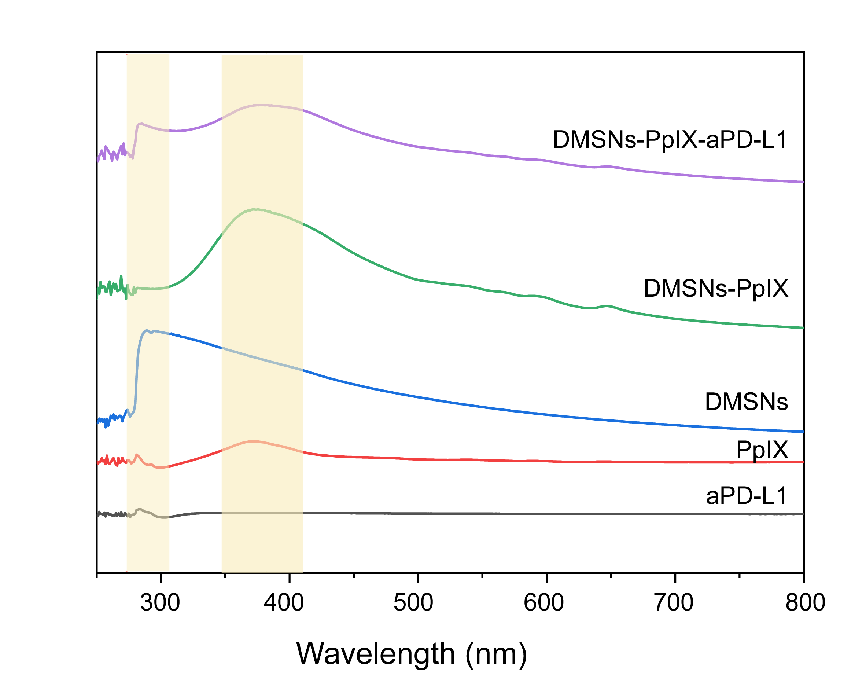


Figure S4. UV-vis analysis of aPD-L1, PpIX, DMSNs, DMSNs-PpIX, and DMSNs-PpIX-aPD-L1.

**
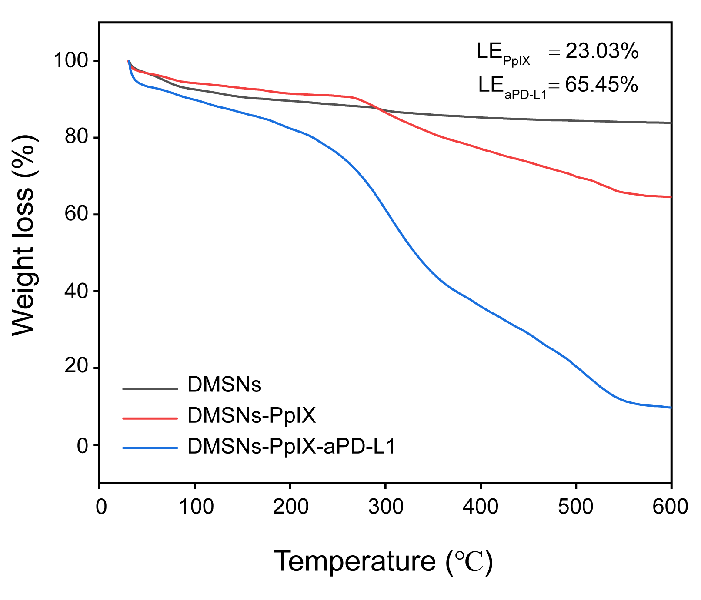
**

Figure S5. thermogravimetric analysis of DMSNs-PpIX and DMSNs-PpIX-aPD-L1.


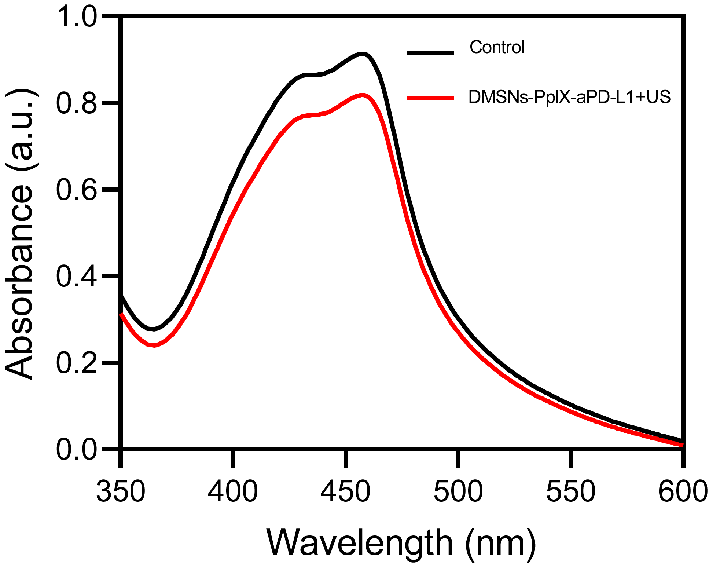


Figure S6. Absorption changes of DPBF after treated with DMSNs-PpIX-aPD-L1+US.


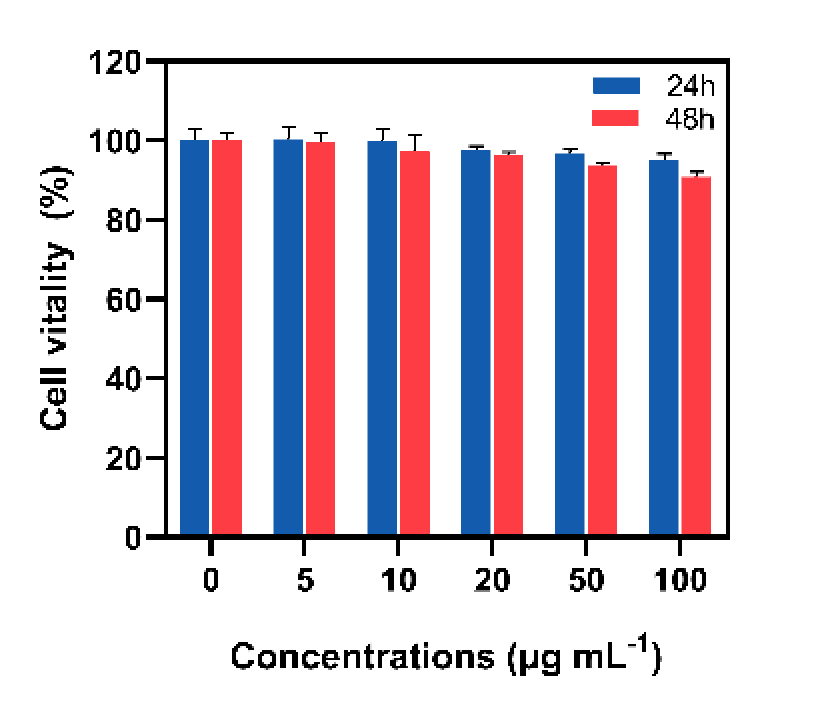


Figure S7. CCK-8 of DMSNs-PpIX-aPD-L1 with L929 cells at different times.


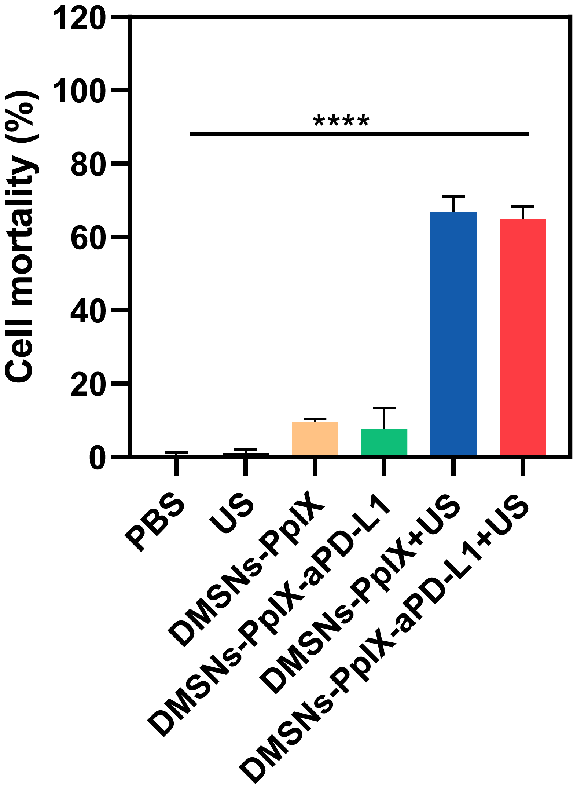


Figure S8. Corresponding quantification of mean fluorescence intensity of Calcein/PI inside cells (n=3) ****p < 0.0001.


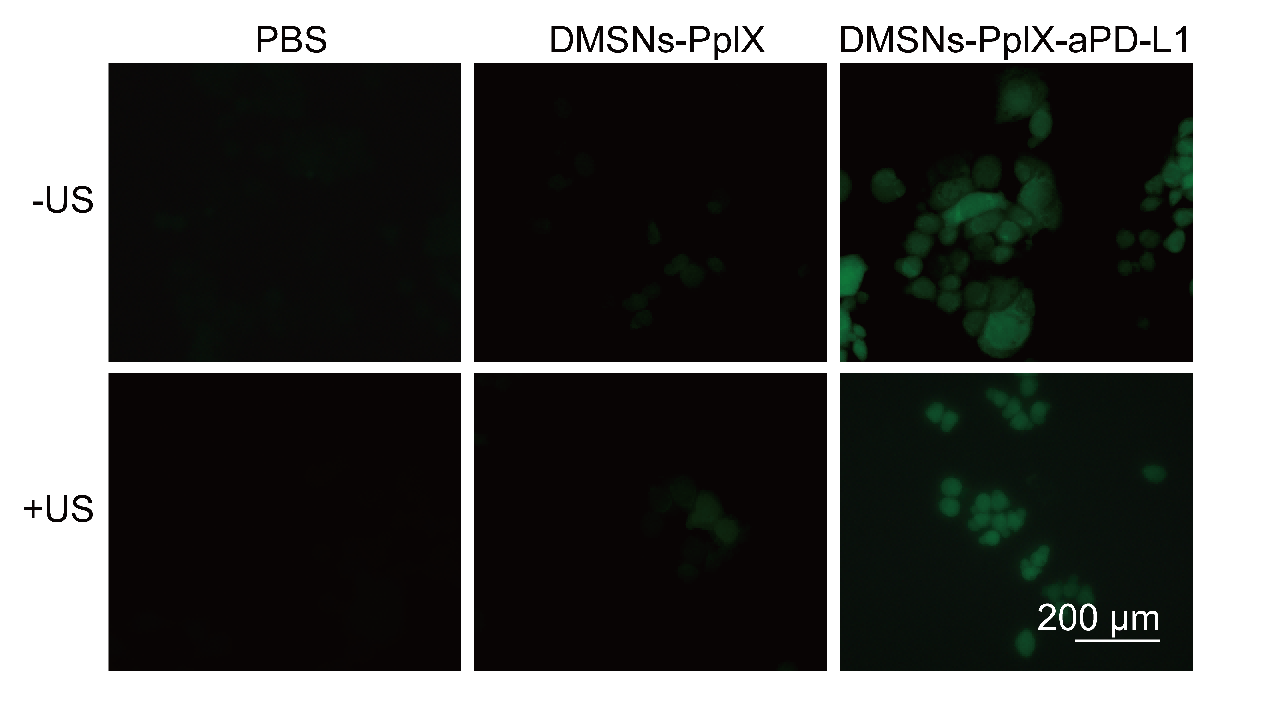


Figure S9. Microscopic images of DCFH-DA-stained 4T1 cells after different treatments.


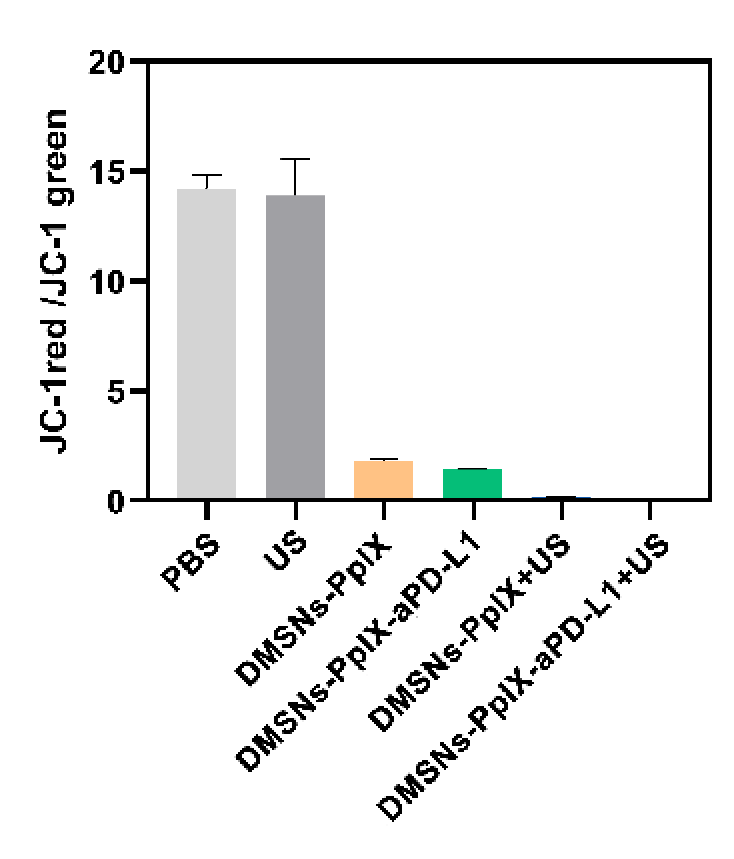


Figure S10. Corresponding quantification of mean fluorescence intensity of JC-1 Aggregates/monomer inside 4T1 cells (n=3).


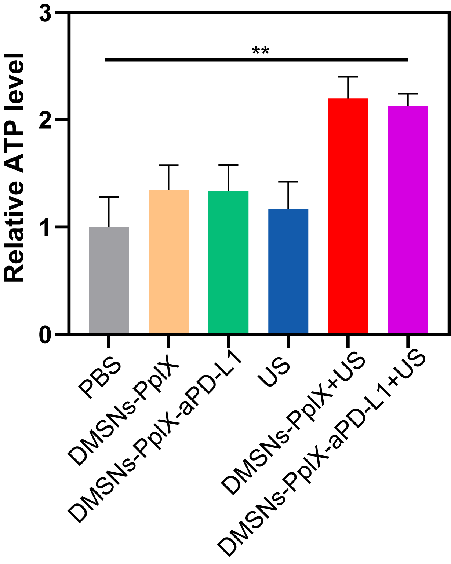


Figure S11. Relative exocellular ATP level after various treatments. **p < 0.01.


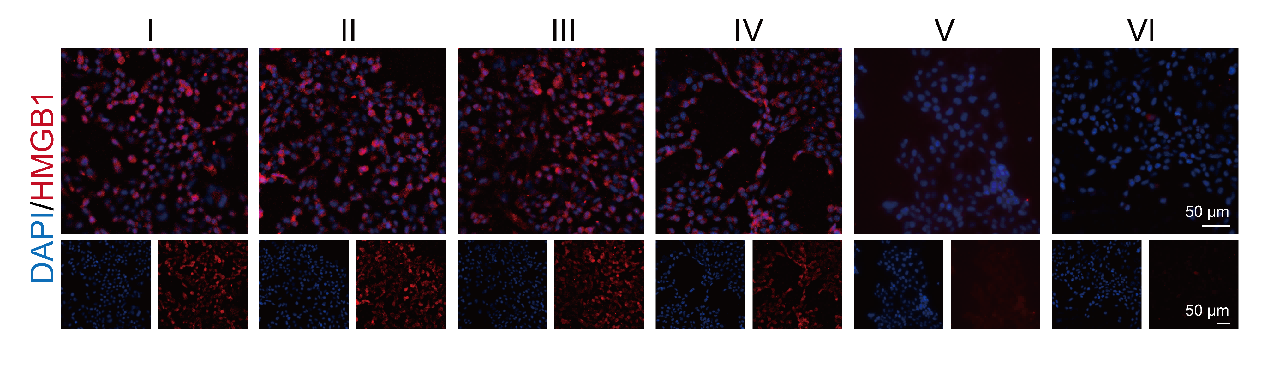


Figure S12. Florescence imaging of HMGB1 after various treatments. Groups: I. PBS; II. US; III. DMSNs-PpIX; IV. DMSNs-PpIX-aPD-L1; V. DMSNs-PpIX+US; VI. DMSNs-PpIX-aPD-L1+US.


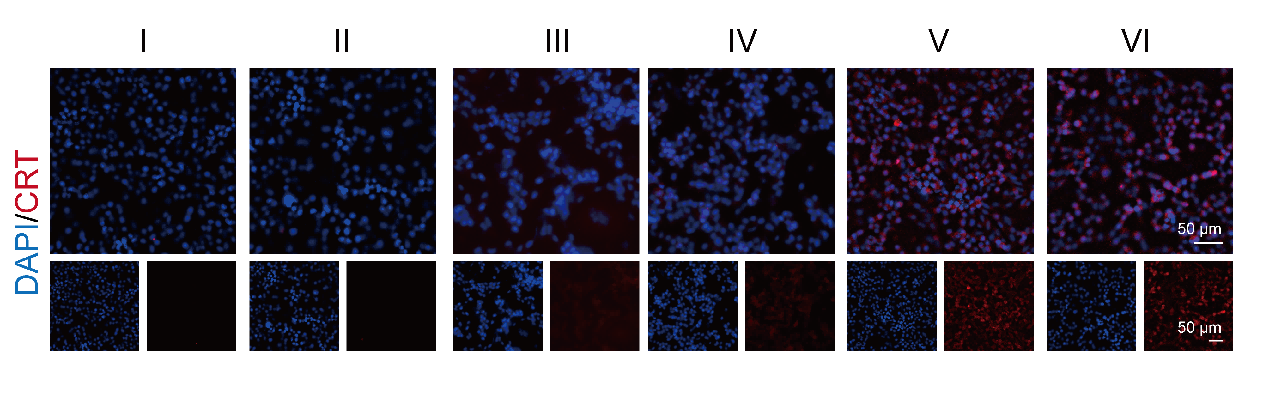


Figure S13. Florescence imaging of CRT after various treatments. Groups: I. PBS; II. US; III. DMSNs-PpIX; IV. DMSNs-PpIX-aPD-L1; V. DMSNs-PpIX+US; VI. DMSNs-PpIX-aPD-L1+US.


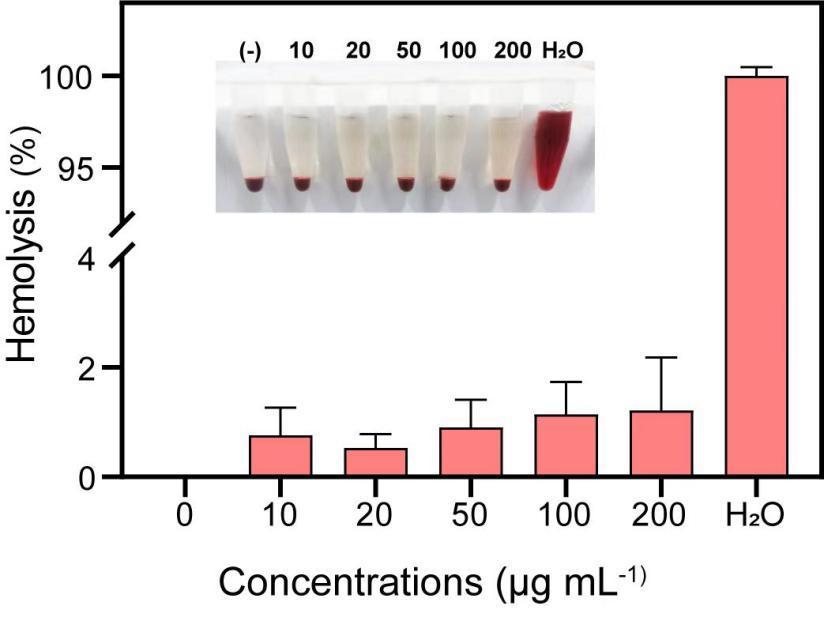


Figure S14. Hemolytic study of DMSNs-PpIX-aPD-L1.


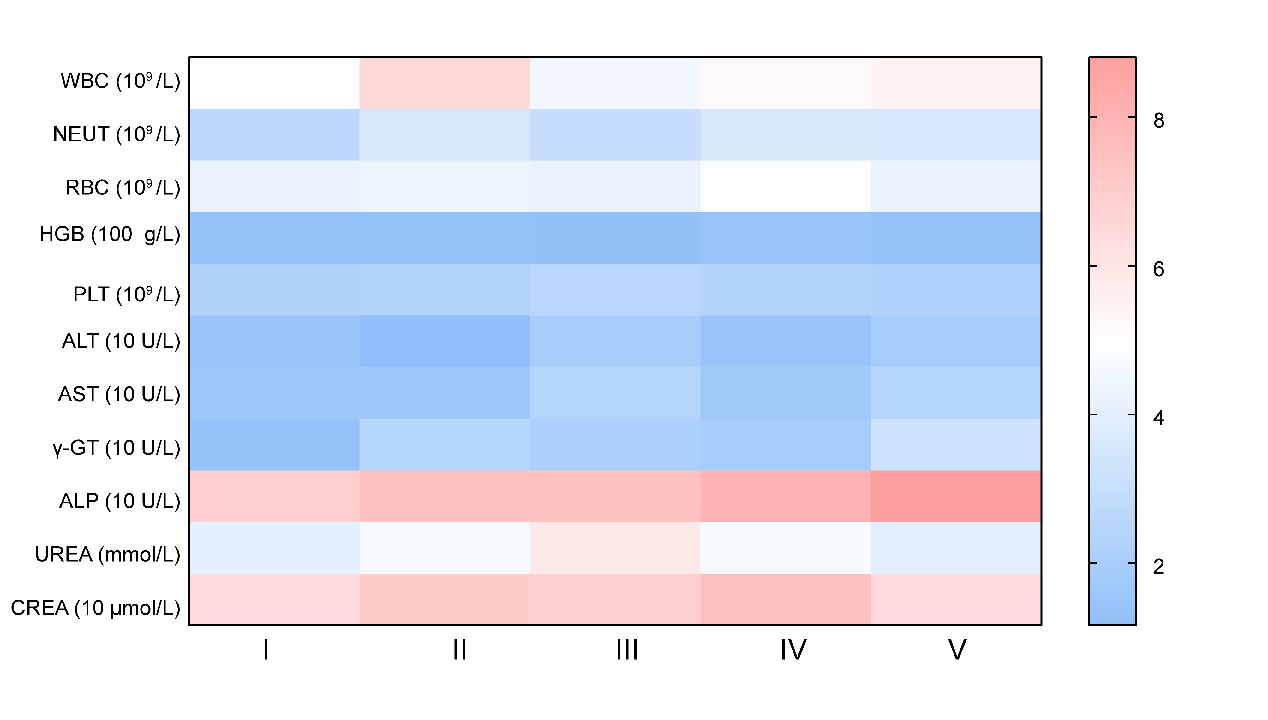


Figure S15. The blood biochemistry and hematology data of mice after different treatments. Groups: I. PBS; II. DMSNs-PpIX; III. DMSNs-PpIX-aPD-L1; IV. DMSNs-PpIX+US; V. DMSNs-PpIX-aPD-L1+US.


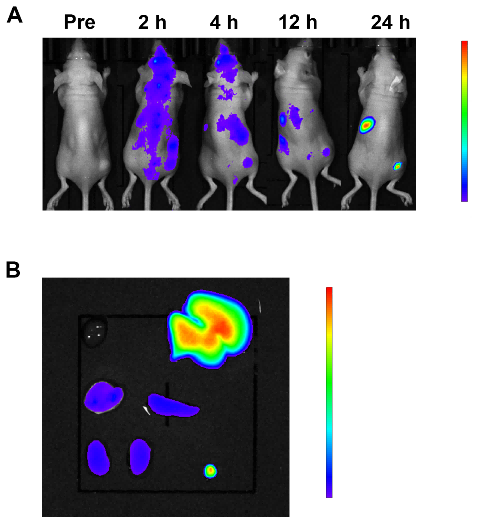


Figure S16. A) Fluorescence images of tumor-bearing mice after tail vein injection of DMSNs-PpIX-aPD-L1 at different time intervals; B) Ex vivo fluorescence imaging of heart, liver, spleen, lung, kidney, and tumor tissues of tumor-bearing mice 24 h after tail vein injection of DMSNs-PpIX-aPD-L1.


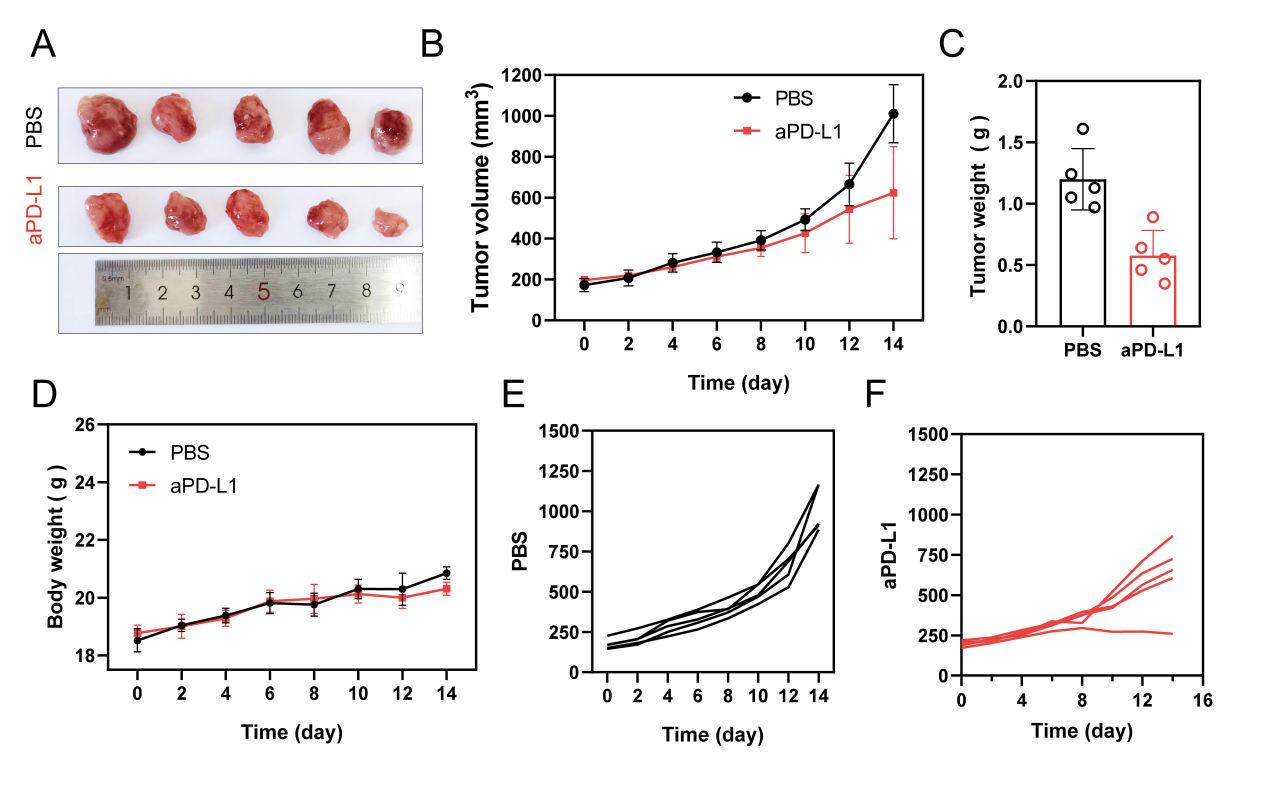


Figure S17. In vivo evaluation of anticancer efficacy of aPD-L1 monotherapy. A) Images of 4T1 tumors excised on day 14 after the treatments. B) Average tumor volume. C) Average tumor weight. D) Average body weight. E) and F) Growth progression of individual tumors.


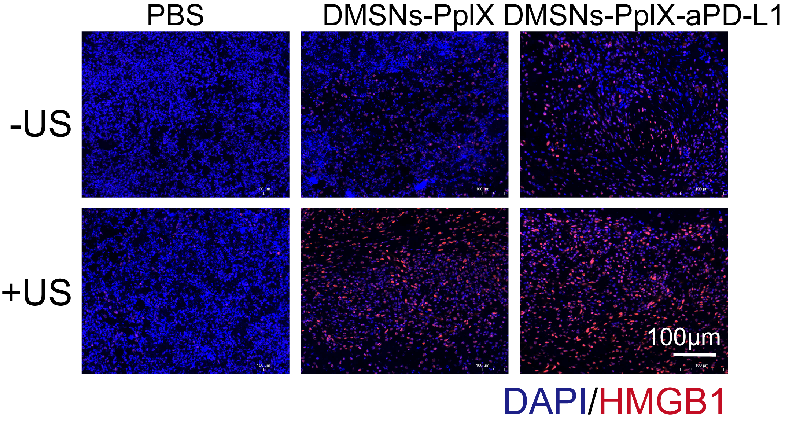


Figure S18. Immunofluorescence images of HMGB1 in the tumor after various treatments.


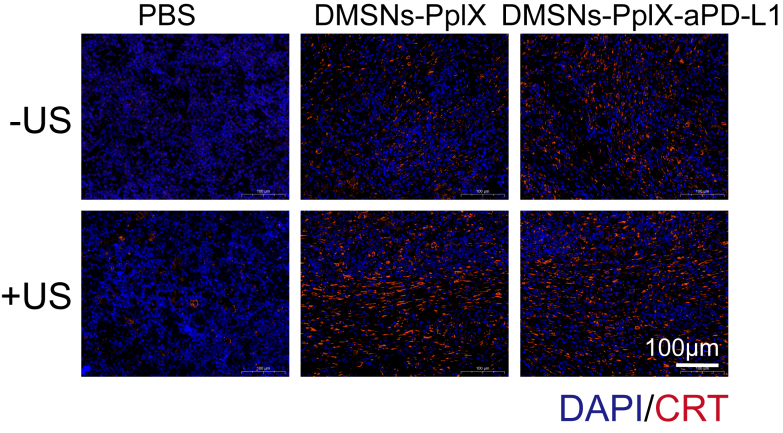


Figure S19. Immunofluorescence images of CRT in the tumor after various treatments.


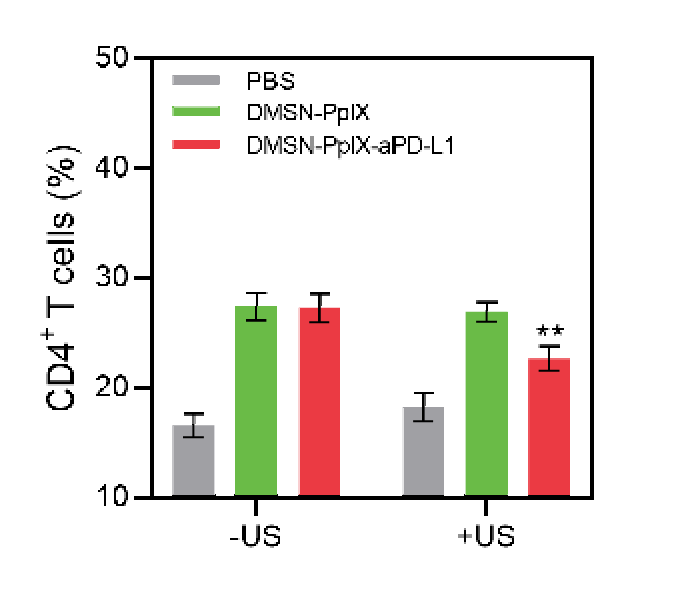


Figure S20. Enumeration of CD4^+^ T cell populations.


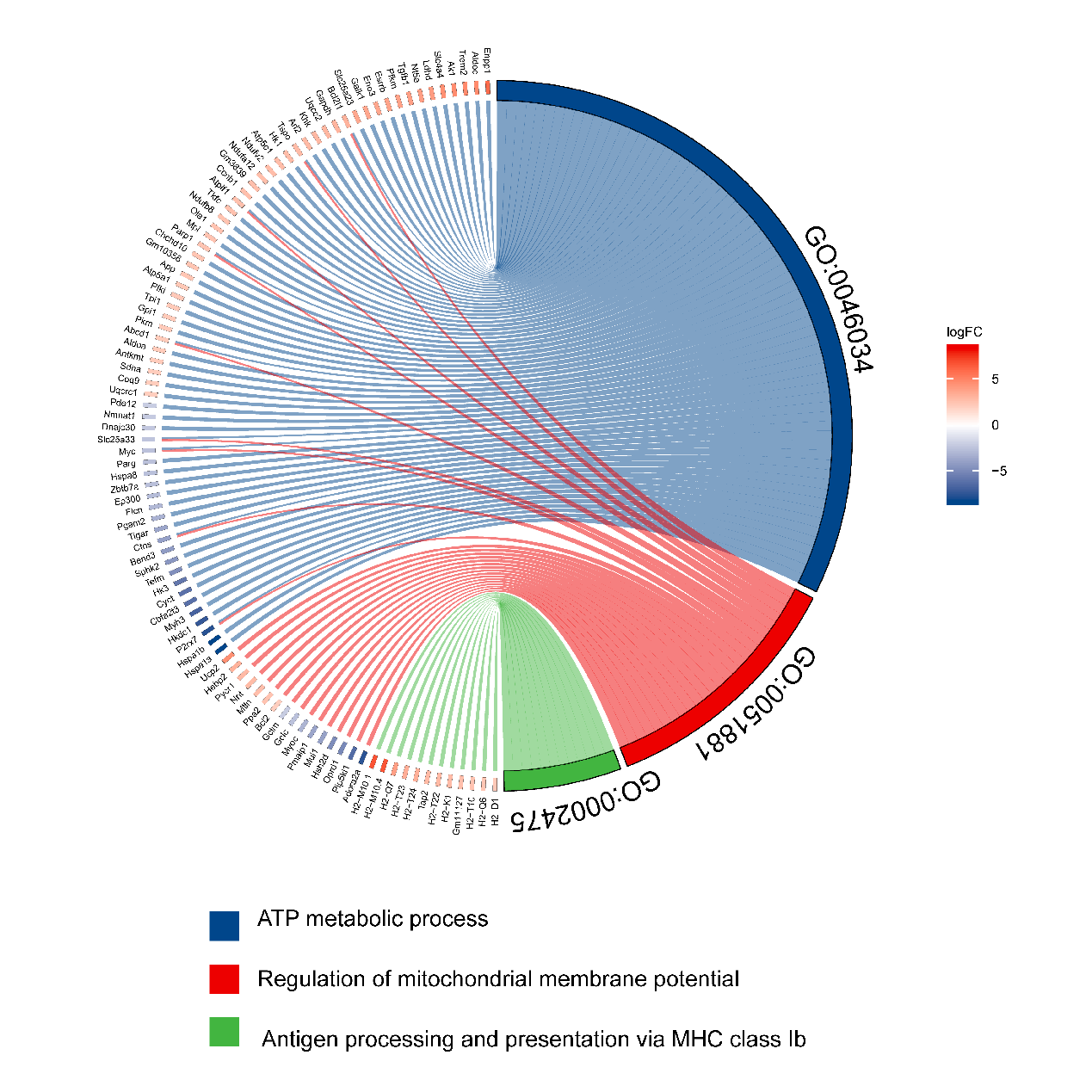


Figure S21. Enriched chord diagram of the GO pathways.


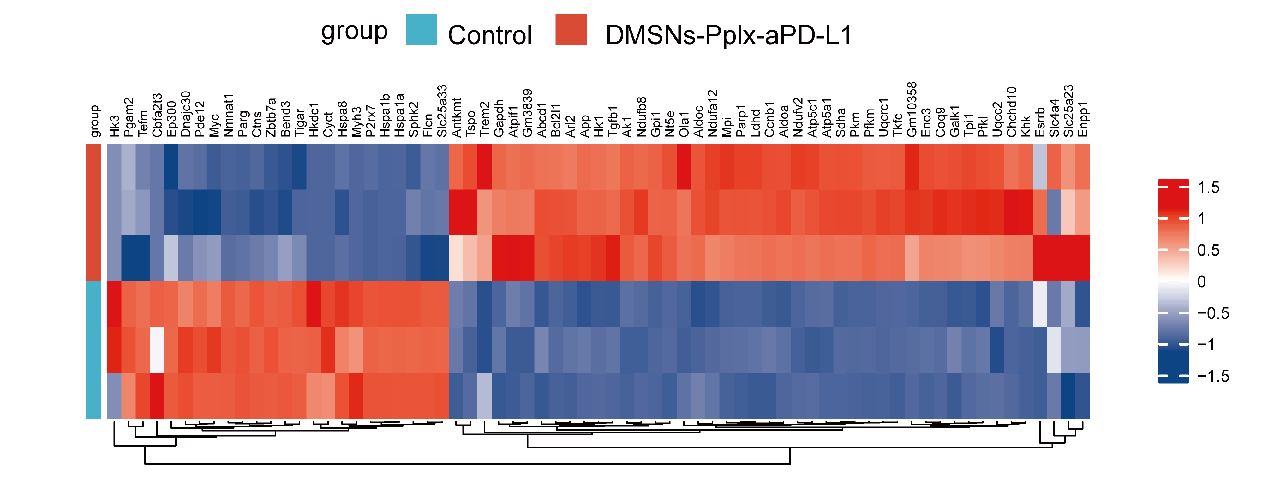


Figure S22. Heatmap of differentially expressed genes (DEGs) involved in the ATP metabolic process pathway in the DMSNs-PpIX-aPD-L1+US and Control groups.
